# Single-cell tumor heterogeneity landscape of hepatocellular carcinoma: unraveling the pro-metastatic subtype and its interaction loop with fibroblasts

**DOI:** 10.1186/s12943-024-02062-3

**Published:** 2024-08-02

**Authors:** De-Zhen Guo, Xin Zhang, Sen-Quan Zhang, Shi-Yu Zhang, Xiang-Yu Zhang, Jia-Yan Yan, San-Yuan Dong, Kai Zhu, Xin-Rong Yang, Jia Fan, Jian Zhou, Ao Huang

**Affiliations:** 1 Department of Liver Surgery and Transplantation, Liver Cancer Institute, Zhongshan Hospital, Fudan University, Key Laboratory of Carcinogenesis and Cancer Invasion (Fudan University), Ministry of Education, 136 Yi Xue Yuan Road, Shanghai, 200032 China; 2 Shanghai Key Laboratory of Organ Transplantation, Zhongshan Hospital, Fudan University, Shanghai, 200032 China; 3 Department of Radiology, Zhongshan Hospital, Shanghai Institute of Medical Imaging, Fudan University, Shanghai, 200032 China; 4 Institute of Biomedical Sciences, Fudan University, Shanghai, 200032 China; 5 State Key Laboratory of Genetic Engineering, Fudan University, Shanghai, 200032 China

**Keywords:** Tumor heterogeneity, Single-cell, Hepatocellular carcinoma, Metastasis, Fibroblasts

## Abstract

**Background:**

Tumor heterogeneity presents a formidable challenge in understanding the mechanisms driving tumor progression and metastasis. The heterogeneity of hepatocellular carcinoma (HCC) in cellular level is not clear.

**Methods:**

Integration analysis of single-cell RNA sequencing data and spatial transcriptomics data was performed. Multiple methods were applied to investigate the subtype of HCC tumor cells. The functional characteristics, translation factors, clinical implications and microenvironment associations of different subtypes of tumor cells were analyzed. The interaction of subtype and fibroblasts were analyzed.

**Results:**

We established a heterogeneity landscape of HCC malignant cells by integrated 52 single-cell RNA sequencing data and 5 spatial transcriptomics data. We identified three subtypes in tumor cells, including ARG1^+^ metabolism subtype (Metab-subtype), TOP2A^+^ proliferation phenotype (Prol-phenotype), and S100A6^+^ pro-metastatic subtype (EMT-subtype). Enrichment analysis found that the three subtypes harbored different features, that is metabolism, proliferating, and epithelial-mesenchymal transition. Trajectory analysis revealed that both Metab-subtype and EMT-subtype originated from the Prol-phenotype. Translation factor analysis found that EMT-subtype showed exclusive activation of SMAD3 and TGF-β signaling pathway. HCC dominated by EMT-subtype cells harbored an unfavorable prognosis and a deserted microenvironment. We uncovered a positive loop between tumor cells and fibroblasts mediated by SPP1-CD44 and CCN2/TGF-β-TGFBR1 interaction pairs. Inhibiting CCN2 disrupted the loop, mitigated the transformation to EMT-subtype, and suppressed metastasis.

**Conclusion:**

By establishing a heterogeneity landscape of malignant cells, we identified a three-subtype classification in HCC. Among them, S100A6^+^ tumor cells play a crucial role in metastasis. Targeting the feedback loop between tumor cells and fibroblasts is a promising anti-metastatic strategy.

**Supplementary Information:**

The online version contains supplementary material available at 10.1186/s12943-024-02062-3.

## Introduction

Hepatocellular carcinoma (HCC), the most common form of primary liver cancer, poses a significant global health burden with its high incidence and mortality rate [1, 2]. The complex and diverse molecular nature of HCC between different tumors and even within the same tumor, manifesting as tumor heterogeneity, presents a formidable challenge in understanding the underlying mechanisms contributing to tumor progression and metastasis [3, 4]. Previous studies have investigated the tumor heterogeneity of HCC using various omics and established several tumor subgroup classifications [5–7]. However, due to technological limitations, these classifications of heterogeneity could not distinguish between tumor cells and tumor microenvironments. Although several studies have investigated the cellular tumor heterogeneity across varies malignancies [8–10], the tumor heterogeneity of HCC in single-cell level remains unclear. Unraveling the intricacies of tumor cell heterogeneity has become pivotal for advancing our understanding of the disease and developing targeted therapeutic strategies.

Metastasis represents the advanced stage of malignancy, usually indicating an incurable disease and the main reason for tumor-related death [11]. Although many studies have investigated the molecules and underlying mechanisms that play vital roles in metastasis [12, 13], the features of tumor cells with a high metastasis potential remain unclear, posing an obstacle to the development of anti-metastasis treatment in HCC. In addition, strong evidence has proven the vital importance of the tumor microenvironment in promoting the metastatic potential of tumor cells [14, 15]. Hence, we aim to comprehensively define and depict the tumor cells with high metastasis potential and reveal the underlying mechanisms of interaction between tumor cells and the tumor microenvironment (TME).

Here, we established the heterogeneity landscape of HCC tumor in the single-cell level, and identified three subtypes of HCC malignant cells. Among them, the S100A6^+^ tumor cells, designated EMT-subtype, have exhibited a strong association with metastasis, indicating a potential key player in the aggressive behavior of HCC. Furthermore, our research reveals an intriguing interplay between the EMT-subtype cells and fibroblasts. The positive feedback loop between the tumor cells and fibroblasts suggests a collaborative role in fostering a pro-metastatic milieu. This novel exploration into the heterogeneous landscape of HCC tumor cells not only refines our understanding of the intrinsic properties of the tumor but also opens avenues for universal intervention strategies. By dissecting the intricate feedback loop between tumor cells and fibroblasts, we provide a new potential target to impede HCC metastasis.

## Results

### Three subtypes in HCC tumor cell heterogeneity landscape

**Fig. 1:**
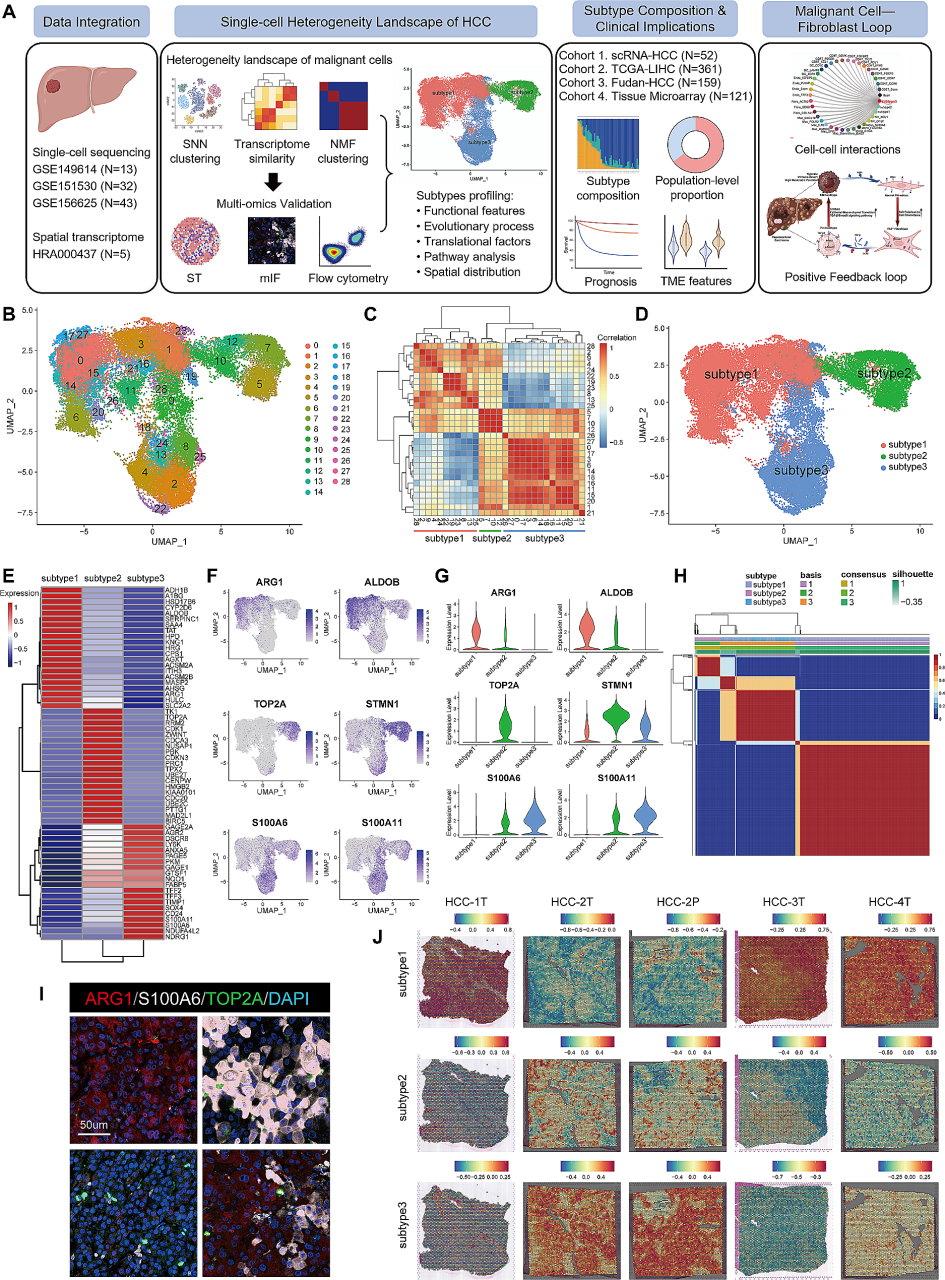
Single cell heterogeneity landscape of HCC identifying three subtypes of tumor cells. (**A**)The scheme for discovering and validating functional tumor subpopulations in HCC. (**B**) The UMAP plot of all tumor cells. (**C**) The heatmap showing the transcriptome correlation between malignant subclusters. (**D**) The UMAP plot showing the three subtypes of tumor cells. (**E**) The heat map showing top highly variable genes of the three subtypes of tumor cells. (**F**) The UMAP plot showing the expression of marker genes of three subtypes in tumor cells. (**G**) The violin plot showing the expression of marker genes of three subtypes in tumor cells. (**H**) The non-negative matrix factorization clustering of tumor cells validating the robustness of three-subtype classification. (**I**) Multiplexed immunofluorescence showing three subtypes of tumor cells, using antibodies ARG1, TOP2A, and S100A6. (**J**) The spatial distribution of three subtypes of tumor cells in spatial transcriptome data

To investigate the heterogeneity landscape of HCC in single-cell level, we initially performed basic analysis for the integrated single-cell RNA (scRNA) sequencing data of HCC tumor samples from three datasets, GSE149614 (*n* = 13) [16], GSE151530 (*n* = 32) [17], and GSE156625 (*n* = 43) [18] (Fig. 1A; Fig. S1A-C). By utilizing the marker genes of HCC tumor cells, such as albumin (ALB) [19] and aldolase 2 (ALDOB) [20], we distinguished the malignancy clusters from immune cells and stromal cells (Fig. S1D-E). The inferred copy number variation (CNV) profile also validated the presence of tumor cells (Fig. S2). Samples with tumor cells fewer than 20 were excluded. Finally, a total of 92,411 cells, including 35,981 tumor cells from 52 HCC samples were included for further analysis (scRNA-HCC cohort; Table S1). Subsequently, re-clustering was performed for the tumor cells, revealing 29 subclusters after eliminating batch effects using the harmony algorithm (Fig. 1B). The UMAP plot illustrated that these subclusters primarily aggregated into three main clusters. To reveal the transcriptome similarity of these subclusters, we computed spearman correlation coefficients between the 29 clusters using the expression profiles of their top 50 genes. Consistent with the UMAP visualization, hierarchical clustering also classified these subclusters into the same main clusters, denoted as subtype1 (subcluster 0,1,3,6,11,14,15,16,17,18,20,21,26,27), subtype2 (subcluster 5,7,10,12), and subtype3 (subcluster 2,4,8,9,13,19,22,23,24,25,28) (Fig. 1C and D). We further identified the highly variable genes (HVGs) specific to each subtype (Fig. 1E; Table S2). The top HVGs of subtype1 were mainly genes associated with metabolism, such as arginase 1 (ARG1) [21] and ALDOB [22]. The top HVGs of subtype2 included genes indicative of proliferation, such as DNA topoisomerase II alpha (TOP2A) [23] and stathmin 1 (STMN1) [24]. The top HVGs of subtype3 comprised S100 calcium binding protein A6 (S100A6) and A11 (S100A11), which actively contribute to tumor progression and metastasis [25, 26] (Fig. 1F and G).

To assess the robustness of this subtype classification, we further conducted non-negative matrix factorization (NMF) clustering for the malignant cells. Firstly, 50 cells from each sample were randomly retrieved to build the matrix. Consistently, non-supervised clustering categorized the malignancy cells into three groups, with the metagenes corresponding to the HVGs identified in the three-subtype classification (Fig. 1H; Fig. S3). More importantly, the results of NMF clustering demonstrated high concordance with the subtype classification (88.2%). Then, we tested the stability of the results of NMF clustering by multiple sampling. Ten times sampling demonstrated that the median concordance rate was 87.4% (range from 86.1 to 88.6%), indicating the robustness of the three-subtype classification system.

To substantiate the existence of these three tumor cell subtypes, multiplexed immunofluorescence (mIF) of ARG1, TOP2A, and S100A6 was performed. mIF confirmed their presence in HCC tissue microarray (TMA), and also illustrated the exclusion expression of these three markers in HCC tumor cells, aligning with the findings from the scRNA-HCC cohort (Fig. 1I). Additionally, we also validated the classification in spatial transcriptome (ST) data (HRA000437) [27]. We found that HCC-1T, 3T and 4T had a high subtype1 score, while HCC-2T and 2P showed a high subtype3 score; the subtype2 score exhibited scattered upregulation across tumor sections of all five ST slides (Fig. 1J). Collectively, these data indicate that HCC tumor cells can be stratified into three distinct subtypes, characterized by the expression of ARG1, TOP2A, and S100A6, respectively.

### The functional features and evolutionary process of three tumor cell subtypes of HCC

**Fig. 2:**
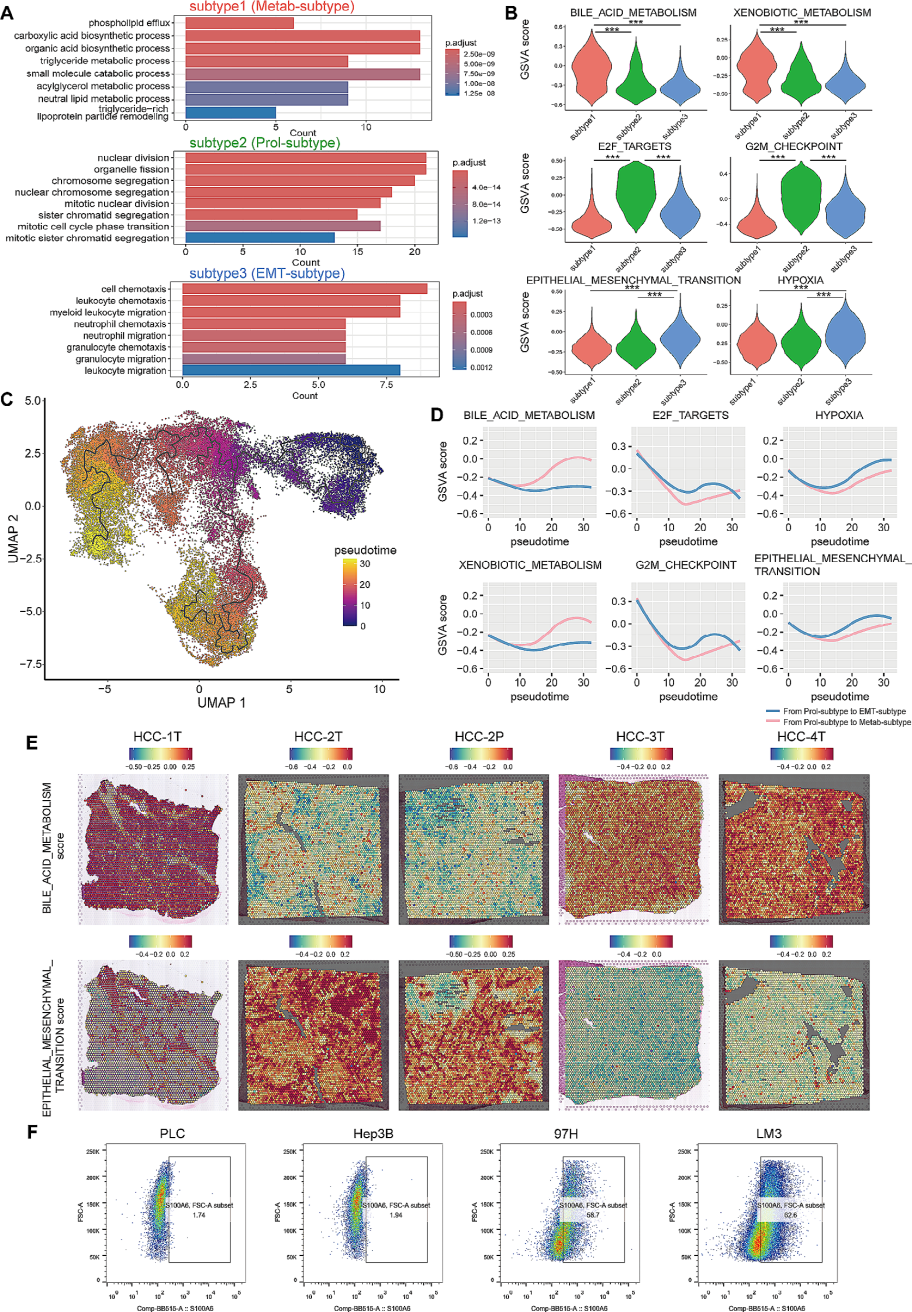
The functional features and evolutionary process of three HCC tumor cell subtypes. (**A**) Bar chart showing the enrichment of specific biological process, based on the highly variable genes of three subtypes of tumor cells. (**B**) Violin plot showing hallmark scores stratified by the three subtypes. (**C**) Evolutionary trajectory of tumor cells, with each color coded for pseudo-time. (**D**) Gene set variation analysis scores of selected hallmarks along pseudotime from Prol-phenotype to Metab-subtype (red line) and from Prol-phenotype to EMT-subtype (blue line). (**E**) The spatial distribution of selected hallmarks scores in spatial transcriptome data. (**F**) Flow cytometry showing S100A6 expression level in four HCC cell lines. PLC, PLC/PRF/5 cells; 97 H, MHCC97H cells; LM3, HCCLM3 cells

To depict the features of the three HCC tumor cell subtypes, we conducted a functional analysis using HVGs of the three subtypes, revealing distinct enrichment patterns in biological processes for each subtype (Fig. 2A). Subtype1 demonstrated significant enrichment in metabolism processes. Consistently, gene set variation analysis (GSVA) indicated higher scores in metabolism-related hallmarks, such as bile acid metabolism and xenobiotic metabolism (Fig. 2B). Thus, we designated subtype1 as the “metabolism subtype” (Metab-subtype). The HVGs of subtype2 were enriched in cell cycle and proliferation. It also exhibited heightened scores of proliferation-related hallmarks including G2M checkpoint and E2F targets (Fig. 2B), leading us to designate it as the “proliferation phenotype” (Prol-phenotype). Notably, the most enriched processes for subtype3 were cell chemotaxis and migration. And it displayed the highest scores in hallmarks of epithelial-mesenchymal transition (EMT) and hypoxia (Fig. 2B), indicating its high metastatic potential and earning it the designation of “EMT subtype” (EMT-subtype).

Intriguingly, we also found that the cancer stem cells (CSC) score, calculated using common CSC markers (EPCAM [28], CD24 [29], KRT19 [30], SOX9 [31], PROM1 [32], CD44 [33], THY1 [34], CD47 [35]), was significantly higher in the EMT-subtype (Fig. S4A & S4B). CSC markers were predominantly expressed in the EMT-subtype, consistent with its ability to develop metastasis (Fig. S4C).

To explore the association between bulk-based HCC classifications [36–38] and our scRNA-seq-based classification, we calculated the score of all these subclasses in tumor cells from 52 HCC samples using ssGSEA. It was observed that Boyault’s G1-G3 subclasses and Hoshida’s S1 and S2 subclasses, representing proliferative tumors [36–39], were both enriched in the EMT-subtype (Fig. S4D). Boyault’s G5-G6 and Hoshida’s S3 subclass, representing well-differentiated tumors, were enriched in the Metab-subtype (Fig. S4D). These results demonstrate consistency between bulk and single-cell classifications. However, we also noted that the EMT-subtype and Prol-phenotype were not clearly distinguished in these bulk-based classifications, highlighting the superiority of single-cell classification in revealing tumor heterogeneity and evolution trajectory at the single-cell resolution.

To clarify the evolutionary relationship between the three subtypes, trajectory analysis was performed, revealing that both the Metab-subtype and the EMT-subtype originated from the Prol-phenotype (Fig. 2C). As expected, along the trajectory from the Prol-phenotype to Metab-subtype, the expression levels of ARG1 and ALDOB were elevated, as well as the scores of metabolism-related hallmarks (Fig. 2D; Fig. S5A). On the contrary, with the evolution from Prol-phenotype to EMT-subtype, the expression levels of S100A6 and S100A11 were elevated, as well as the scores of EMT and hypoxia hallmarks (Fig. 2D; Fig. S5A). In addition, TOP2A and KI67 was strikingly downregulated during the trajectory, as well as the proliferation-related hallmarks (Fig. 2D; Fig. S5A).

We subsequently validated the features in ST data. It was observed that the ST sections with a high Metab-subtype score (HCC-1T, 3T, 4T) exhibited increased scores of metabolism hallmarks, while those with a high EMT-subtype score (HCC-2T, 2P) showed correspondingly elevated EMT and hypoxia scores (Fig. 2E; Fig. S5B).

### Validation of the single-cell classification in cell lines

Validation of the subtype classification was extended to HCC cell lines. Firstly, we retrieved the single-cell RNA sequencing data of HUH7 and MHCC97-H (97H) from GSE188289 (Fig. S6A & S6B). The GSVA scores of the three subtypes were subsequently calculated, and the tumor cells were assigned accordingly (Fig. S6C-S6E). It revealed the exclusive presence of Metab-subtype cells in HUH7 cell line and EMT-subtype cells in 97H cell line (Fig. S6E & S6F). Additionally, it demonstrated the presence of Prol-phenotype subpopulations in both HUH7 and 97H cell lines, further confirming that the Prol-phenotype represents cells in a proliferative state (Fig. S6E). Notably, we also found the Prol-phenotype cells from the 97H cell line showed significantly higher EMT-subtype and lower Metab-subtype scores compared to Prol-phenotype cells from the HUH7 cell line, indicating that the proliferating cells maintained their original features (Fig. S6G). The metastatic potential of HUH7 and 97H were demonstrated using migration and invasion assays, which was consistent with their single-cell classification subtyping (Fig. S6H).

Considering all cell lines had subpopulations of Prol-phenotype, we further obtained expression data of 31 commercial cell lines from GSE97098 and classified these cell lines based on the expression patterns of Metab-subtype and EMT-subtype (Fig. S6I). Among the 31 cell lines, 9 were assigned to the Metab-subtype and 22 were assigned to the EMT-subtype. Furthermore, the expression patterns of Hep3B, 97H and HCCLM3 (LM3) from another dataset (GSE49994) validated Hep3B as Metab-subtype and 97H as EMT-subtype cell line (Fig. S6J). The LM3 was also classified as EMT-subtype (Fig. S6J).

To further substantiate the classification, we performed flow cytometry to assess the expression of marker genes in four HCC cell lines with different metastatic potential (high metastatic potential: 97H, LM3; low metastatic potential: Hep3B, and PLC/PRF/5 (PLC)). Consistent with the analysis based on single-cell transcriptome data, all cell lines contained a subpopulation expressing the proliferation markers KI67 (Fig. S7A). 97H and LM3 exhibited increased S100A6 expression, and decreased ARG1 expression, thus classified as the EMT-subtype (Fig. 2F; Fig. S7B). In contrast, Hep3B and PLC displayed reduced S100A6 expression and elevated ARG1 expression, identified as the Metab-subtype (Fig. 2F; Fig. S7B). These alignment findings validated the robustness of the single-cell classification and underscored the EMT-subtype tumor cell marked by S100A6 as the subtype with high metastatic potential.

### Transcription factors profile revealing activated SMAD3 and TGF-β signaling pathway in EMT-subtype

**Fig. 3:**
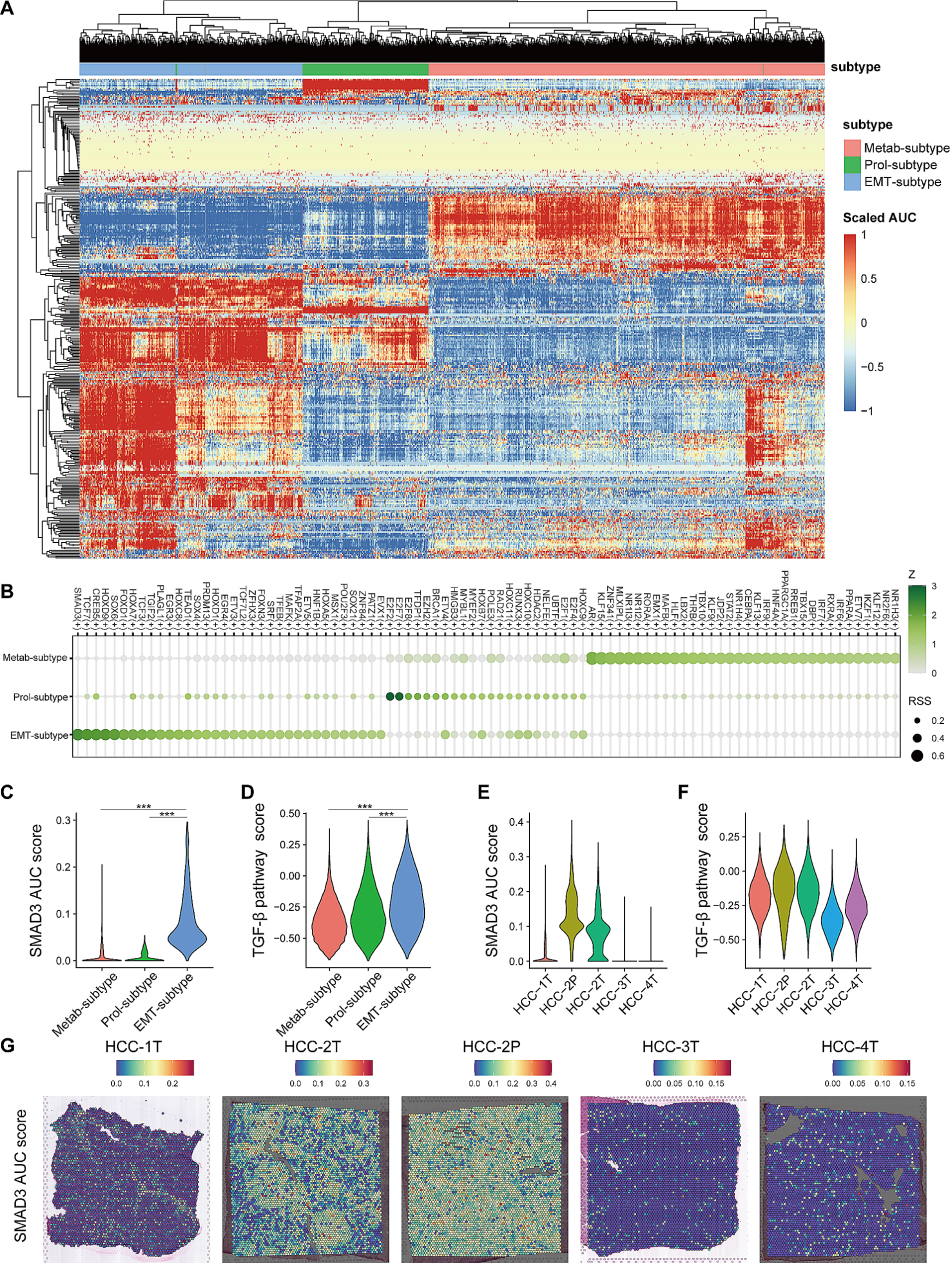
The transcription factors profile of three HCC tumor cell subtypes. (**A**) The heat map of the area under the curve (AUC) scores of translation factors (TF) in HCC tumor cells. (**B**) The top TFs of the three subtypes of tumor cells, ranked by the regulon specificity score. (**C**) SMAD3 AUC score stratified by the three subtypes. (**D**) TGF-β pathway score stratified by the three subtypes. (**E**) SMAD3 AUC score stratified by sample in spatial transcriptome data. (**F**) TGF-β pathway score stratified by sample in spatial transcriptome data. (**G**) The spatial distribution of SMAD3 AUC score in spatial transcriptome data

To decode the underlying mechanisms of the distinct subtypes, we conducted pySCENIC analysis to explore the activated transcription factors (TFs) in the three subtypes. To attenuate the effects of noise and outliers, pseudo-cells were calculated as an average of 20 cells randomly selected from the same subtype and applied for the analysis [40, 41]. The activities of TFs were quantifying by the Area Under the Recovery Curve (AUC) for genes regulated by each TF. Notably, the TFs landscape also clustered tumor cells into three groups, closely aligning with the identified subtypes, thereby further substantiating the robustness of the subtype classification (Fig. 3A). TF specific to each subtype were also sorted according to the regulon specificity score (RSS) respectively (Fig. 3B). For the Metab-subtype, the top activated TFs included NR1I3, NR1I2, which are involved in drug metabolism [42] and energy metabolism [43], and HNF4A, a fatty acid metabolism-related TF [44] (Fig. 3B; Fig. S8B & S8C). The Prol-phenotype exhibited activation of cell cycle-related TFs, including E2F2 and E2F7 [45]. Importantly, the EMT-subtype was characterized by the exclusive activation of SMAD3 in the TGF-β-SMAD pathway [46], and TCF7 in WNT pathway [47], with SMAD3 displaying the highest RSS among the TFs (Fig. 3B and C). GSVA analysis corroborated the upregulation of the TGF-β signaling pathway in the EMT-subtype (Fig. 3D), consistent with its established function of promoting metastasis in the advanced stage tumors [48, 49].

Similar observations were discerned in the ST data, where TGF-β pathway was upregulated in HCC-2T and 2P (Fig. 3F; Fig. S8A). Consistently, only these two sections displayed pronounced activation of SMAD3 (Fig. 3E and G). In the contrast, the HCC-1T, 3T, and 4T showed reduced TGF-β signaling and diminished SMAD3 activation, while exhibiting activation of NR1I3, NR1I2, and HNF4A, consistent with the results from scRNA-HCC cohort (Fig. S8D-S8F). However, TCF7 wasn’t identified as activated in HCC-2T and 2P. Collectively, these results underscored that TGF-β-SMAD pathway, specifically SMAD3, might play a core role in the formation of EMT-subtype.

Additionally, similar observations were discerned in the HCC cell lines. The 97H and LM3 displayed significantly upregulated SMAD3 phosphorylation compared with Hep3B and PLC (Fig. S7C).

### The subtype composition, sample-level heterogeneity, and its clinical implications

**Fig. 4:**
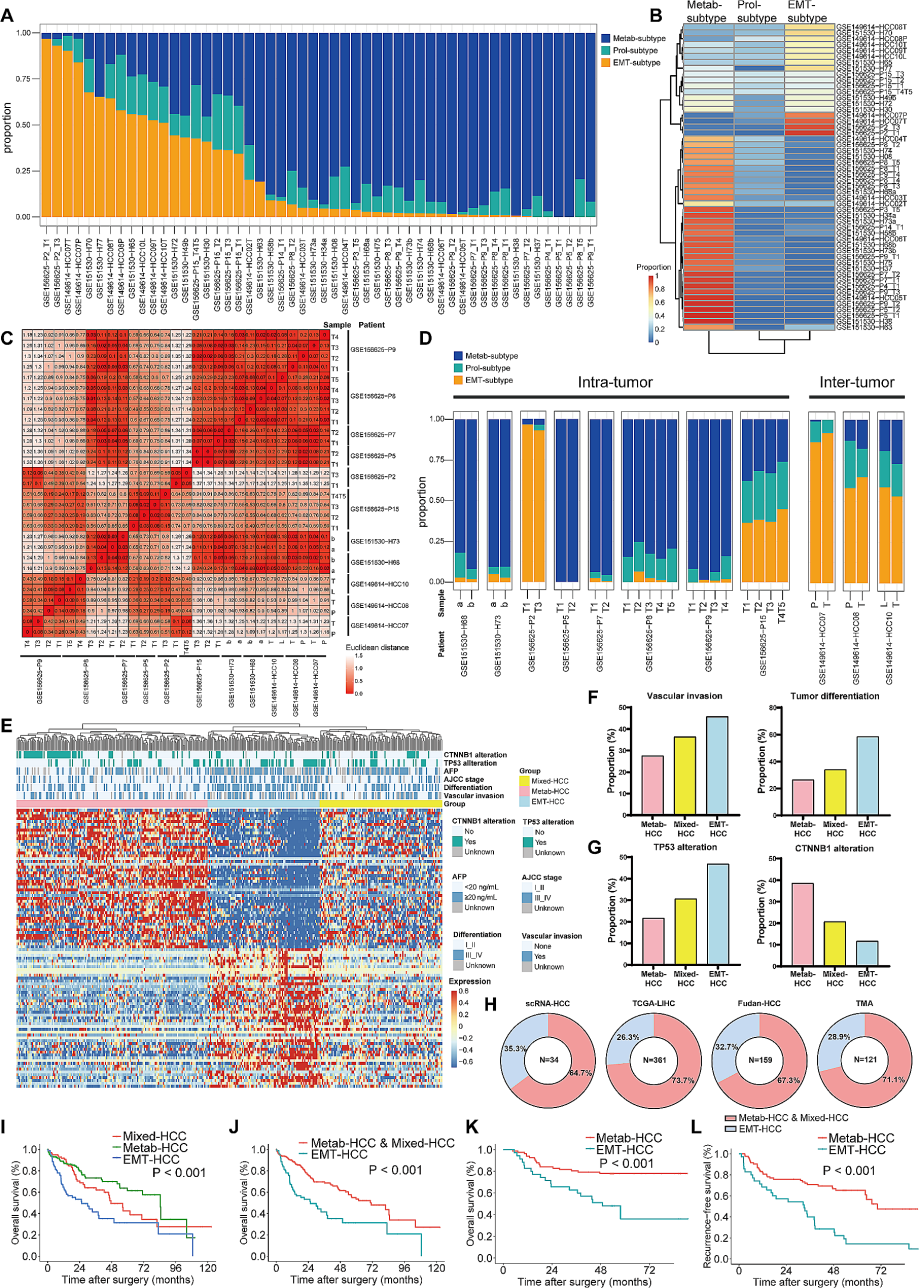
The composition of three subtypes in sample-level. (**A**) The bar chart showing the composition of the three subtypes of tumor cells in each sample from the scRNA-HCC cohort. (**B**) The heat map showing two groups of HCC based on the composition of the three subtypes. (**C**) The heat map showing the Elucidation distance between multiple sampling tumors within the same patient based on the composition of the three subtypes. (**D**) The bar chart showing the composition of the three subtypes in multiple sampling tumors within the same patient. (**E**) The top 50 highly variable genes of Metab-subtype and EMT-subtype divided HCC tumors into three subgroups (Metab-HCC, EMT-HCC, and Mixed-HCC) in the TCGA-LIHC cohort. (**F**) The vascular invasion and tumor differentiation in Metab-HCC, Mixed-HCC, and EMT-HCC. (**G**) The mutation rates of TP53 and CTNNB1 in Metab-HCC, Mixed-HCC, and EMT-HCC. (**H**) The proportion of EMT-HCC in the population across four cohorts, respectively. (**I**) Kaplan-Meier curves for overall survival (OS) stratified by the three subgroups of HCC in the TCGA-LIHC cohort. (**J**) Kaplan-Meier curves for OS of EMT-HCC in the TCGA-LIHC cohort. (**K**) Kaplan-Meier curves for OS of EMT-HCC in the TMA cohort. (**L**) Kaplan-Meier curves for recurrence-free survival of EMT-HCC in the TMA cohort

To elucidate the composition of the three tumor cell subtypes in bulk HCC samples, we computed the proportion of each subtype within HCC samples. Intriguingly, the majority of samples exhibited a stable but not dominant proportion of Prol-phenotype cells, while Metab-subtype and EMT-subtype cells predominated in mutually exclusive HCC samples (Fig. 4A). Consequently, hierarchical clustering categorized samples as either HCC dominated by Metab-subtype cells (Metab-HCC) or HCC dominated by EMT-subtype cells (EMT-HCC) (Fig. 4B). In total, 33 samples were designated as Metab-HCC, and 19 samples were classified as EMT-HCC.

Analysis of patients with multi-sampling underscored that tumor samples from the same patient exhibited a similar subtype composition (Fig. 4C). The median Euclidean distance between tumor samples within one patient was 0.079 (range: 0-0.200), which was significantly lower than it between different patients with a median value of 0.671 (range: 0.009–1.369) (*P* < 0.001). Specifically, samples obtained from the same tumor, whether sampled simultaneously (19 samples from GSE156625) or at different time points (4 samples from GSE151530), displayed analogous proportions (Fig. 4D). Moreover, primary tumor and metastatic tumor from the same patient (6 samples from GSE149614; T for primary tumor, *P* for portal vein tumor thrombus (PVTT), and L for metastatic lymph node) also demonstrated similar composition (Fig. 4D). Notably, the EMT-subtype dominated in all patients with metastasis, both in primary and metastatic tumors, reinforcing the association between EMT-subtype and metastasis. Additionally, the ST data also showed the same phenomenon. The HCC-1T, 3T, and 4T was defined as Metab-HCC, while HCC-2T and HCC-2P (the PVTT of HCC-2T) was defined as EMT-HCC (Fig. 1J).

Furthermore, we validated the subtypes in two bulk transcriptome datasets of HCC, the TCGA-LIHC cohort [6] and the Fudan-HCC cohort [5]. The top 50 HVGs of the Metab-subtype and EMT-subtype were utilized in hierarchical clustering. Concordantly, in the TCGA-LIHC cohort, the heatmap illustrated that patients could be classified as Metab-HCC, EMT-HCC, and Mixed-HCC (expressing HVGs of both Metab-subtype and EMT-subtype) (Fig. 4E). The vascular invasion risk and tumor differentiation worsened from the Metab-HCC, through Mixed-HCC to EMT-HCC (Fig. 4F). There was increasing mutation rates of TP53 while decreasing mutation rates of CTNNB1 from Metab-HCC to EMT-HCC. (Fig. 4G). Remarkably, the three groups of HCC exhibited distinct outcomes, with a median overall survival (OS) of 84.4 months in Metab-HCC, 47.4 months in Mixed-HCC, and 25.5 months in EMT-HCC (Fig. 4I). Patients with EMT-HCC experienced significantly poorer outcomes compared to those with Metab-HCC or Mixed-HCC (median OS: 25.5 vs. 70.5 months; *P* < 0.001; Fig. 4J).

We validated the correlation between mutation and subtype in cell lines. Cell lines belonging to the EMT-subtype exhibited a TP53 mutation rates of 72.7% (16/22), slightly higher than that in cell lines of the Metab-subtype (55.6%, 5/9). In contrast, the Metab-subtype show a CTNNB1 mutation rate of 22.2% (2/9), while the EMT-subtype showed 9.1% (2/22). Although the statistical significance was limited due to the small sample size, this trend supports the correlation between mutation and subtype at the single-cell level.

In addition, we validated the clinical implications of subtype classification in the Fudan-HCC cohort. Consistently, tumors could be delineated as Metab-HCC, EMT-HCC, or Mixed-HCC (Fig. S9A), which also demonstrated different OS and recurrence-free survival (RFS) (Fig. S9B-S9C). Patients with EMT-HCC exhibited a significantly poorer OS (median: 21.8 months vs. not reached; *P* < 0.001; Fig. S9D) and RFS (median: 12.2 months vs. 39.7 months; *P* = 0.008; Fig. S9E) than those with Metab-HCC or Mixed-HCC. Additionally, the mIF on TMA distinguished Metab-HCC and EMT-HCC based on the expression of ARG1 and S100A6, which also confirmed the shorter OS (median: 48.7 months vs. not reached, *P* < 0.001, Fig. 4K) and RFS (median: 33.1 vs. 70.1 months, *P* < 0.001, Fig. 4L) of EMT-HCC.

Besides, we investigated the percentage of EMT-HCC in four cohorts. The demographic characteristics and etiologies of the four cohorts were presented in Table S3. It was revealed that EMT-HCC constituted 35.3%, 26.3%, 32.7%, and 28.9% of HCC patients in the scRNA cohort, TCGA-LIHC cohort, Fudan-HCC cohort, and TMA cohort, respectively (Fig. 4H), showing a consistent share among patients from different cohorts.

### EMT-HCC harbored a desert microenvironment featured with fewer infiltration of CD8^+^T and NK cells

Subsequently, we explored the ecosystem features of EMT-HCC. NK and T cells were re-clustered according to previous article [50, 51] (Fig. S10A; Fig. S11A & S11B). EMT-HCC exhibited a significantly diminished presence of NK cells and CD8^+^ T cells but an elevated abundance of CD4^+^ FOXP3^+^ regulatory T (Treg) cells (Fig. S11C). Most subclusters of NK cells and CD8^+^ T cells showed a decrease in proportion in the microenvironment of EMT-HCC (Fig. S11D). Immunohistochemistry (IHC) images revealed that EMT-HCC had significantly fewer infiltrations of CD56^+^ NK cells and CD8^+^ T cells but an enrichment of CD4^+^ FOXP3^+^ Treg cells (Fig. S11E). Additionally, myeloid cells were re-clustered accordingly [52, 53] (Fig. S11F; Fig. S10B). We identified an absence of FOLR2^+^ macrophages and IL1B^+^ macrophages in EMT-HCC (Fig. S11G & S11H), which were reported to be associated with CD8^+^ T cell infiltration in tumors [53]. In EMT-HCC, we found an enrichment of SPP1^+^ macrophages, a modulator of the microenvironment in metastasis [54, 55], although the difference was not statistically significant (Fig. S11H).

### EMT-subtype tumor cells recruited fibroblasts and educated it to FAP^+^ fibroblasts via SPP1-CD44 ligand-receptor pair

**Fig. 5:**
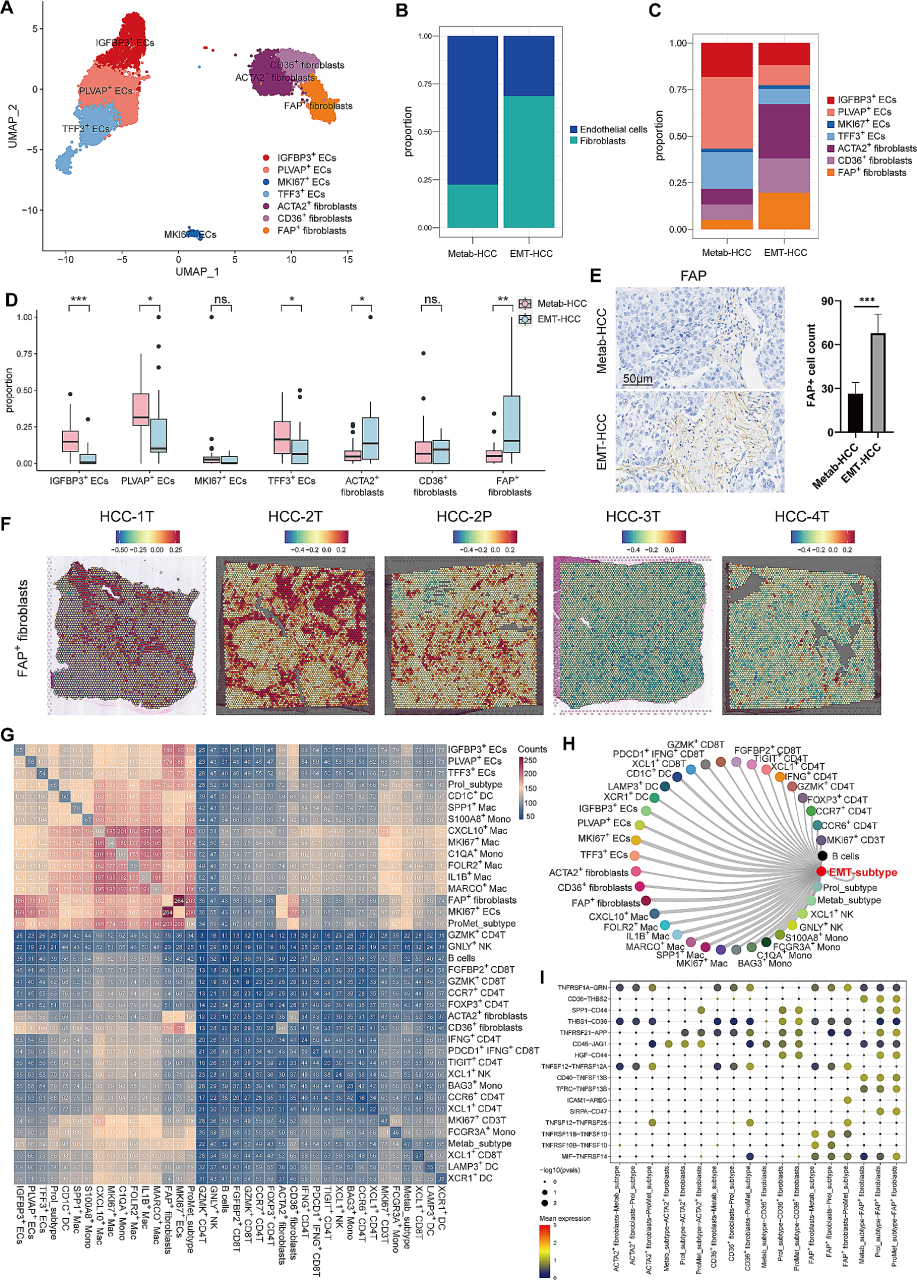
The activation of fibroblasts in EMT-HCC. (**A**) The UMAP plot of stromal cells. (**B**) Stacked bar chart showing the compositions of endothelial cells and fibroblasts in Metab-HCC and EMT-HCC. (**C**) Stacked bar chart showing the compositions of subclusters of endothelial cells and fibroblasts in Metab-HCC and EMT-HCC. (**D**) Box plot showing the fraction of subclusters of endothelial cells and fibroblasts in Metab-HCC and EMT-HCC. (**E**) Immunohistochemistry images showed the number of FAP^+^ fibroblasts in Metab-HCC and EMT-HCC. (**F**) The spatial distribution of FAP^+^ fibroblasts in spatial transcriptome data. (**G**) The heat map showing numbers of inferred ligand-receptor pairs between all cell types. (**H**) Network plots showing inferred ligand-receptor interaction activity between EMT-subtype and other cell types. (**I**) Dot plot showing selected interactions pairs between tumor subtypes and fibroblasts

As for the stromal cells, we observed a striking enrichment of fibroblasts in EMT-HCC, while endothelial cells were markedly reduced (Fig. 5A and B). After re-clustering stromal cells into subclusters [56, 57] (Fig. S10C), we identified a significant enrichment of FAP^+^ fibroblasts in EMT-HCC (Fig. 5C and D). These findings were further validated in the TMA cohort. IHC demonstrated an increased presence of FAP^+^ fibroblasts in EMT-HCC (Fig. 5E). Additionally, validation using the ST data showed a significantly enrichment of FAP^+^ fibroblasts in HCC-2T compared to Metab-HCC sections. Intriguingly, even the PVTT in HCC-2P exhibited substantial FAP^+^ fibroblasts enrichment (Fig. 5F).

To investigate the interactions between tumor subtypes and TME, a cell-cell interaction analysis was conducted. Intriguingly, we observed that EMT-subtype tumor cells showed the most active interactions with FAP^+^ fibroblasts (203 receptor-ligand pairs), while Metab-subtype cells only had half the number of interaction pairs (Fig. 5G and H). These results indicated that EMT-subtype cells and FAP^+^ fibroblasts had substantial impacts on each other, consistent with previous studies [58, 59]. After scrutinizing the ligand-receptor pairs, we found that EMT-subtype cells secreted many cytokines, such as SPP1, HGF, THBS1, and MIF (Fig. 5I). Among them, we identified that SPP1-CD44 was exclusively presented in EMT-subtype cells rather than Metab-subtype cells. Meanwhile, it had an impact not only on FAP^+^ fibroblasts but also on ACTA2^+^ fibroblasts and CD36^+^ fibroblasts (Fig. 5I), indicating that the SPP1-CD44 might be the specific ligand-receptor pair for fibroblasts activation. Thus, we focused on the SPP1-CD44 between EMT-subtype and fibroblasts in the further experiments.

**Fig. 6:**
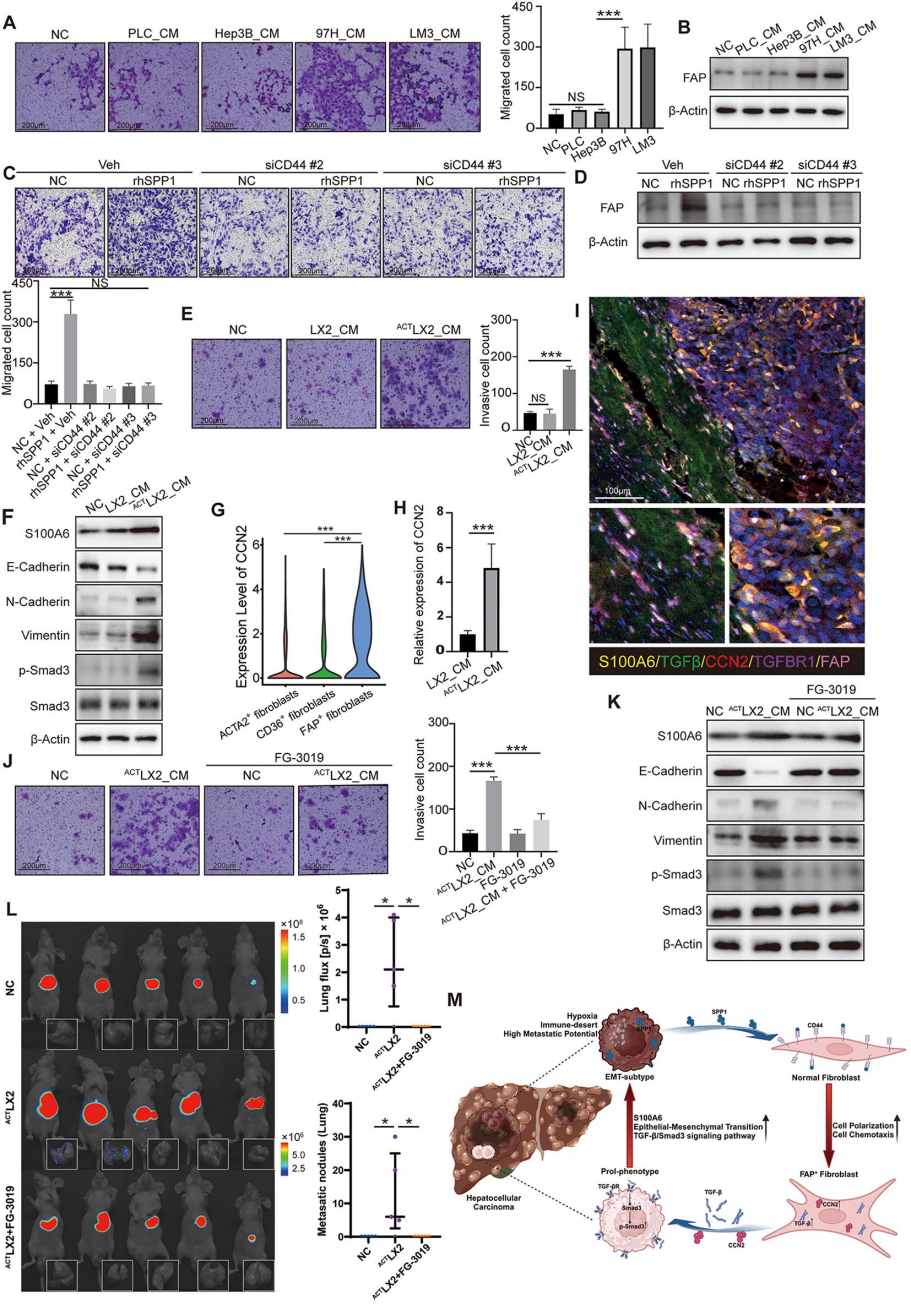
The interaction loop of SPP1-CD44 and CCN2/TGFβ-TGFBR1 between tumor cells and fibroblasts. (**A**) Transwell assay showed that the conditioned medium of 97H and LM3 significantly recruited LX2 cells. (**B**) Western blotting assay showed that the conditioned medium of 97H and LM3 significantly promoted the protein level of FAP in LX2 cells. (**C**) Transwell assay showed that rhSPP1 significantly promoted the recruitment of LX2 cells, while siCD44 reversed this change. (**D**) Western blotting showed that rhSPP1 significantly promoted the protein level of FAP in LX2 cells, which was reversed by siCD44. (**E**) Transwell assay showed that ^ACT^LX2-CM significantly promoted the invasive ability of PLC cells. (**F**) Western blotting assay showed that ^ACT^LX2-CM significantly increased the expression levels of S100A6, N-cadherin, Vimentin and p-Smad3 while decreased the expression levels of E-cadherin in PLC cells. (**G**) The expression of CCN2 in fibroblasts subclusters from scRNA-HCC cohort. (**H**) ELISA assay showed high levels of secreted CCN2 within ^ACT^LX2-CM. (**I**) Multiplexed immunofluorescence images showing the interaction between malignant cells and fibroblasts, based on the CCN2/TGF-β-TGFBR1, using antibodies S100A6, CCN2, TGF-β, TGFBR1, and FAP. (**J**) Transwell assay showed that FG-3019 reversed the enhanced invasion capacity of PLC cells induced by ^ACT^LX2-CM. (**K**) Western blotting assay showed that FG-3019 reversed the up-regulated expression of S100A6, N-cadherin, Vimentin, and p-Smad3 and down-regulated expression of E-cadherin induced by ^ACT^LX2-CM. (**L**) Bioluminescence images of liver tumors (top) and lung metastasis (bottom) of in situ tumor transplantation model. *n* = 5 for each group. (**M**) Schematic diagram of the feedback loop between malignant cells and fibroblasts. NC, Normal Control; PLC, PLC/PRF/5 cells; 97H, MHCC97H cells; LM3, HCCLM3 cells; CM, conditioned medium; Veh, siRNA control vehicle; siCD44, CD44 sensitive siRNA; rhSPP1, recombinant human protein SPP1; ^ACT^LX2-CM, Conditioned medium of SPP1-pretreated LX2 cells

We extracted conditioned medium (CM) from four HCC cell lines cultured in vitro (PLC-CM, Hep3B-CM, 97H-CM, LM3-CM) to stimulate LX2 cells, a hepatic stellate cell line, to mimic the effects of tumor cells on fibroblasts in vivo. Transwell assay revealed that 97H-CM and LM3-CM recruited more LX2 cells than PLC-CM and Hep3B-CM (Fig. 6A). Besides, 97H-CM and LM3-CM enhanced expression of FAP protein in LX2 cells, inducing the switch to FAP^+^ fibroblasts (Fig. 6B). After confirming the enhanced expression of SPP1 in 97H and LM3 cells than PLC and Hep3B cells (Fig. S12A). We knocked down SPP1 in 97H cells(^siSPP1^97H) using siRNA (Fig. S12B) and extracted their conditioned medium (^siSPP1^97H-CM) to stimulate LX2 cells. ^siSPP1^97H-CM lost the ability to recruit LX2 cells (Fig. S12C). Similarly, ^siSPP1^97H-CM could not promote the LX2 cells to FAP^+^ fibroblasts (Fig. S12D). Next, we stimulated LX2 cells with recombinant human SPP1 protein (rhSPP1) while CD44-sensitive siRNA (siCD44) to block their binding to receptor. Western blotting showed that siCD44 successfully decreased CD44 expression of LX2 cells (Fig. S12E). It was found that rhSPP1 significantly promoted the recruitment of LX2 cells, whereas siCD44 significantly reversed this phenomenon (Fig. 6C). Western blotting revealed that rhSPP1 educated LX2 cells to FAP^+^ fibroblasts, which was also reversed by siCD44 (Fig. 6D). The above results suggested that SPP1-CD44 receptor-ligand pair was the key signaling mediator to recruit and activate fibroblasts.

### The educated FAP^+^fibroblasts promoted the translation to EMT-subtype by activating the TGF-β-SMAD pathway through the secretion of CCN2

We further investigated the effect of FAP^+^ fibroblasts on tumor cells. Conditioned medium of SPP1-pretreated LX2 cells (^ACT^LX2-CM) was used to stimulate PLC cells. ^ACT^LX2-CM significantly promoted the migration and invasion of PLC cells (Fig. 6E; Fig. S12F), and increased the expression of S100A6 in PLC cells (Fig. 6F). Correspondingly, the expression level of EMT-related proteins such as N-cadherin and vimentin were significantly increased while E-cadherin was decreased (Fig. 6F). Above results suggested that the ^ACT^LX2-CM could transform PLC cells to EMT-subtype.

To investigate which specific subtype of PLC transformed into the EMT-subtype, we employed two different methods to inhibit cell proliferation, including mitomycin C treatment and deprivation of fetal bovine serum in culture medium (Fig. S12G). It was found that inhibiting cell proliferation significantly reversed the upregulation of S100A6 induced by ^ACT^LX2-CM. These results revealed that the translation occurs in proliferating cells (Fig. S12H).

Translation factors analysis has revealed that TGF-β-SMAD pathway, especially SMAD3, played vital role in EMT-subtype (Fig. 3B and D), which was also supported by the activation of SMAD3 in PLC treated with ^ACT^LX2-CM (Fig. 6F). Intriguingly, we found that FAP^+^ fibroblasts had significantly upregulation of CCN2 (Fig. 6G), previously reported as a coactivator of the TGF-β-SMAD pathway via direct binding to TGF-β [60]. Consistently, ELISA assay found ^ACT^LX2-CM harbored enhanced secreted CCN2 compared to the LX2-CM (Fig. 6H). mIF images visualized the spatial proximity of S100A6^+^ TGFBR1^+^ tumor cells and FAP^+^ CCN2^+^ fibroblasts (Fig. 6I). The colocalization of CCN2/TGF-β/TGFBR1 in the membrane of S100A6^+^ tumor cells further confirmed the interaction pair (Fig. 6I).

Then, we stimulated PLC cells with recombinant human TGF-β and CCN2 protein (rhTGF-β and rhCCN2). It was demonstrated that the rhCCN2 enhanced the function of rhTGF-β to promote the migration and invasion ability of PLC cells (Fig. S12I & S12J). Same phenomenon was reflected via the protein expression level of S100A6, E-cadherin, N-cadherin, Vimentin, and Phospho-Smad3 in PLC cell (Fig. S12K).

Next, we added FG-3019, an CCN2 antagonist, in ^ACT^LX2-CM to treat PLC cells. It was revealed that FG-3019 significantly reversed the enhancement of migration and invasion ability of PLC cells caused by ^ACT^LX2-CM (Fig. 6J; Fig. S12L). Similarly, the expression of S100A6, E-cadherin, N-cadherin, Vimentin and Phospho-Smad3 was also reversed by FG-3019 (Fig. 6K). In vivo study also confirmed the inhibition of metastasis via FG-3019. Regular injection of ^ACT^LX2-CM in distant seeding mouse model tail-vein-injected with PLC promoted the incidence of pulmonary metastasis, while co-injection of ^ACT^LX2-CM and FG-3019 diminished the metastasis rates (Fig. S12M). For further validation of the results of in vivo experiments, we constructed two types of subcutaneous tumors (PLC cells single, NC group; PLC and ^ACT^LX2 cells co-injection, ^ACT^LX2 group) and transplant tumor tissue into liver in situ. We discovered more lung metastases in the ^ACT^LX2 group, while injection of FG-3019 inhibit lung metastasis (Fig. 6L). The above results suggested that FAP^+^ fibroblasts might confer enhanced metastatic ability to tumor cells with low metastatic propensity through the TGF-β signaling pathway in vivo, whereas the CCN2 inhibitor significantly blocked this effect in metastatic ability.

## Discussion

In this study, we initiated an exploration into the heterogeneity landscape of malignant cells in HCC, revealing a tripartite classification comprising Metab-subtype (ARG1^+^ tumor cell), Prol-phenotype (TOP2A^+^ tumor cell), and EMT-subtype (S100A6^+^ tumor cell). These three subtypes demonstrated distinct features of metabolism, proliferating, and pro-metastasis respectively. We further unveiled that the Metab-subtype and EMT-subtype represent distinct endpoint statuses originating from the Prol-phenotype. The establishment of the three-subtype classification marks a new investigation into the heterogeneity of tumor in single-cell era. By elucidating the features and evolutions of the three subtypes in HCC, we identified and characterized the tumor cells with high metastatic potential and confirmed S100A6 as the biomarker. More importantly, the ubiquitous presence of the three subtypes of tumor cells across all HCC patients reminds us that there may be common therapeutic targets which could circumvent heterogeneity of HCC.

Although ARG1 has been applied as a diagnostic biomarker for HCC, previous studies have reported that ARG1 serves as a biomarker of well-differentiated HCC [61, 62], and reduced ARG1 expression is associated with a poor prognostic phenotype [63]. Regarding the Prol-phenotype, we defined it as proliferating cells according to several evidence. Firstly, HVGs of this subtype are enriched in cell cycle-related pathways. Secondly, cells of this subtype are consistently present in all samples, suggesting a relatively stable proportion. Lastly, analysis of single-cell data from cell lines reveals the presence of Prol-phenotype subpopulations in both Metab-subtype and EMT-subtype cell lines, further confirming that the Prol-phenotype represents cells in a proliferative state. The definition of Prol-phenotype clarified the relationship and evolutions between different tumor cell subtypes. Furthermore, from the perspective of single cells, we demonstrated that tumor tissues are composed of varying proportions of Metab-subtype and EMT-subtype tumor cells, along with relatively stable proportions of Prol-phenotype tumor cells. These different proportions determine the varying proliferation and metastatic potential of the tumor as a whole.

We observed a significant upregulation of hypoxia and CSC features in EMT-subtype, consistent with the previous studies implicating hypoxia and stemness in promoting tumor metastasis [29, 64, 65]. Further investigation unveiled the activation of the SMAD3 and the TGF-β-SMAD pathway in the EMT-subtype, indicating the pivotal role of this pathway in the context. This finding is supported by the evidence demonstrating that the TGF-β-SMAD pathway can promote metastasis in HCC by inducing EMT [49, 66]. We observed that the proportion of EMT-HCC ranged from 26.3% to 35.3% in patients with HCC. These parts of patients had significantly unfavorable outcomes and a remarkably desert TME. By comprehensively delineating the features of EMT-subtype and EMT-HCC, we provide insights into the molecular mechanisms of tumor cells with high metastatic potential and indicate the direction for anti-metastatic strategies.

Significantly, we discovered an interaction loop between the EMT-subtype and fibroblasts. The heightened expression of SPP1 in the EMT-subtype was found to recruit fibroblasts and induce their activation into FAP^+^ fibroblasts through binding to CD44. Notably, we found that the FAP^+^ fibroblasts secreted CCN2, previously reported as a coactivator of the TGF-β pathway via direct binding to TGF-β [60], cooperating with TGF-β to activate the pathway, thus promoting the transition of tumor cells into the EMT-subtype. Crucially, the inhibition of CCN2 was observed to significantly disrupt the feedback loop between tumor cells and the FAP^+^ fibroblasts, consequently mitigating its potential for tumor metastasis. In the present study, we revealed a novel feedback loop between HCC tumor cells and fibroblasts mediated by the SPP1-CD44 and CCN2/TGF-β-TGFBR1 interaction pairs. This reciprocal interaction and positive feedback loop represent a promising target for anti-metastasis treatment.

Several limitations should be acknowledged. Firstly, the scRNA data and ST data were derived from public datasets. Additional in-house scRNA data are warranted to validate the reliability of the three-subtype classification. Secondly, the ST data has not reached the single-cell resolution. Each spot in the ST data contains an estimate of 1 to 10 cells [67]. Thus, a single-cell resolution ST method is needed for precise depiction of the spatial heterogeneity landscape of tumor cells.

In conclusion, we established a single-cell tumor heterogeneity landscape of HCC and revealed a three-subtype classification of tumor cells. Among them, the S100A6^+^ EMT-subtype harbored a pro-metastatic phenotype characterized by the upregulation of EMT and hypoxia, along with activation of the TGF-β-SMAD pathway. HCCs dominated with EMT-subtype cells displayed a poorer prognosis and a desert TME. Furthermore, we unveiled a feedback loop between tumor cells and fibroblasts, representing a promising anti-metastatic target. Our study provides previous unknown insights into the tumor heterogeneity of HCC and identified a potential target for metastasis.

### Methods

#### scRNA data collection and basic analysis

Three public datasets of single-cell RNA sequencing were obtained from the Gene Expression Omnibus database (GSE149614 [16], GSE151530 [17], and GSE156625 [18]). All HCC tumor samples were retrieved for further integration analysis. For quality control, all cells with fewer than 500 features or a mitochondrial gene fraction above 15% were removed. After quality control, we used the R package harmony (v1.2.0) [68] to integrate the expression data from different samples and used the Seurat (v4.3.0) [69] to perform the basic downstream analysis and visualization. In detail, we first performed normalization, log-transformation, centering, and scaling on them. Next, 2000 highly variable genes were identified and utilized to conducted principal components analysis (PCA) to project the cells into a low-dimensional space. The first 20 principal components were used. Then, by setting the samples as the batch factor and employing the “RunHarmony” function, we iteratively corrected the cells’ low-dimensional PC representation to mitigate the impact of batch effect. The corrected PC matrixes were used to perform unsupervised shared-nearest neighbor–based clustering and UMAP visualization analysis. Differential expression analysis based on clusters was used to identify marker genes and manually annotate clusters based on information from literature.

Sub-clustering of the major cell types was performed by subsetting the cell types of interest and repeated the above process in the subset. After that, manual annotation of clusters based on marker genes was performed and contaminating clusters were removed.

### ST data collection and cell-type estimation

The ST data were obtained from Genome Sequence Archive (HRA000437) [27]. All HCC tumor samples were retrieved for further analysis. We used harmony to integrate the expression data from different sections and used the Seurat to normalize data. ssGSEA [70] in GSVA package (V1.48.3) was utilized to score the cell types in ST spots.

### CNV comparison analysis

The CNVs of tumor cells were estimated on the basis of their transcriptome profiles using the method of infercnv (V1.18.1) [71]. Stromal cells were used as a normal reference.

### Transcriptome similarity analysis

To assess the transcriptome similarity of different tumor cell clusters, we firstly calculated the average expression within each cluster. And then sorted the top 50 genes with highest standard variations among clusters. The similarity among clusters was evaluated using nonparametric Spearman correlation based on the expression profile of the top 50 genes. After that, hierarchical clustering was performed using the correlation coefficients to assigned the clusters into subgroups with similar transcriptome features.

### NMF clustering

NMF analysis was performed using the NMF (V0.26) in downsampled tumor cells (50 cells from each sample) from scRNA data [72], with k = 3 as the number of factors. Three expression patterns of tumor cell and the corresponding metagenes were identified. Unsupervised clustering on these metagenes was then performed using NMFconsensus. To assess the robustness of NMF clustering, we repeated the this analysis 10 times by resampling tumor cells each time.

### Pathway enrichment analysis and gene set variation analysis

We conducted the enrichment analysis for the biology process by using clusterProfiler (V4.8.3) [73]. The HVGs (avg_logFC > 1.0) of each subtype were used in enrichment analysis.

The GSVA analysis were performed on the hallmark pathways collected in the molecular signature database (MSigDB, V2023.2.Hs) [74], and GSVA scores were obtained using the GSVA package (v1.34.0) [75].

### Bulk RNA-seq and survival analysis

We fetched bulk RNA-seq data of patients with HCC from the Cancer Genome Atlas (TCGA-LIHC [6]) and the National Omics Data Encyclopedia (Fudan-HCC [5]). Unsupervised hierarchical clustering was applied to define subgroups of HCC using the top 50 HVGs of Metab-subtype and EMT-subtype. Subsequently, we performed Kaplan–Meier analysis used survival (V3.5-7) and survminer (V0.4.9). The significance was assessed through the log-rank test.

### Cell differentiation trajectory inference

To infer the differentiation trajectory of tumor cells, we used monocle3 (V1.3.4) [76] to infer the pseudotime of each cell using default parameter and DDR-Tree method.

### pySCENIC

Single-cell regulatory network inference and clustering (pySCENIC, V0.12.1) [77] workflow was applied to investigate the regulation strength of transcription factors in tumor cells from scRNA data or tumor spots from ST data. We assessed TF activity by quantifying the AUC for genes regulated by each TF. The RSS of each TF was also calculated. Given the challenges posed by the original pySCENIC implementation in handling large datasets and its susceptibility to sequencing depth effects, we enhanced its scalability and robustness by aggregating data from every 20 randomly selected cells within each cell type. Subsequently, pySCENIC analysis was applied to the average gene expression profile derived from the pooled data [40, 41, 78].

### Sample-level heterogeneity analysis

To analyze the sample-level heterogeneity, we initially selected all patients with simultaneous or heterochronic multi-sampling. The subtype composition of each sample was used to calculate the Euclidean distance between samples. Then, hierarchical clustering was performed for unsupervised clustering of the samples based on the Euclidean distance. Heterogeneity was assessed by the Euclidean distance between samples derived from the same.

### Cell-cell interaction analysis

To analyze cell-cell interactions between different cell types, we used CellphoneDB V4 to infer the significant ligand-receptor pairs within HCC samples [79]. The cell type-specific ligand-receptor interactions between cell types were identified based on the specific expression of a receptor by one cell type and a ligand by another cell type. The interaction score refers to the total mean of the individual ligand-receptor partner average expression values in the corresponding interacting pairs of cell types. We selected the significantly differential ligand–receptor pairs and used cellchat (V1.6.1) to visualize the results [80].

### Statistical analysis

Cell distribution comparisons between groups were performed using unpaired two-tailed Wilcoxon rank-sum tests. Comparisons of gene expression or gene signature between groups of cells were performed using unpaired two-tailed Student’s t test. All statistical analyses and presentation were performed using R software (V4.2.3) or GraphPad Prism software (V9.0). Statistical difference results were demonstrated: ns., not significant; +, *P* < 0.1; *, *P* < 0.05; **, *P* < 0.01; ***, *P* < 0.001.

## Electronic supplementary material

Below is the link to the electronic supplementary material.

Supplementary Material 1

Supplementary Material 2

Supplementary Material 3

Supplementary Material 4

Supplementary Material 5

## Data Availability

No datasets were generated or analysed during the current study.
