# Supplementary material for: Single-cell tumor heterogeneity landscape of hepatocellular carcinoma: unraveling the pro-metastatic subtype and its interaction loop with fibroblasts
Source: Mol Cancer. 2024 Aug 2;23:157. doi: 10.1186/s12943-024-02062-3 (PMC11295380; doi:10.1186/s12943-024-02062-3)
Supplement: Supplementary file 4 — Supplementary Material 4 [file 12943_2024_2062_MOESM4_ESM.docx]

**Supplementary Content**

**Single-Cell Tumor Heterogeneity Landscape of Hepatocellular Carcinoma: Unraveling the Pro-Metastatic Subtype and its Interaction Loop with Fibroblasts**

**Table of contents**

**Supplementary Methods**

- Multiplexed immunofluorescence
- Flow cytometry
- Histopathological analysis and immunohistochemistry Staining
- siRNA transfection
- Cell co-culture
- Transwell assay
- Cell proliferation inhibition assay
- Western blotting
- ELISA assay
- Metastatic model based on tail vein injection
- Metastatic model based on tumor transplantation

**Supplementary Figures**

- Fig. S1. Identifying malignant cells from integrated single-cell RNA sequencing data.
- Fig. S2. The inferred CNV of malignant cells.
- Fig. S3. The summary of NMF clustering results.
- Fig. S4. The distributions of cancer stem cells (CSC) features and bulk-based HCC classifications among the three tumor cell subtypes.
- Fig. S5. The functional features and evolutionary process of three HCC tumor cell subtypes.
- Fig. S6. Validation of the three HCC tumor cell subtypes in cell lines.
- Fig. S7. The expression of marker genes and translation factors in HCC cell lines.
- Fig. S8. The specific activated translation factors and pathways in three subtypes.
- Fig. S9. Validation of three-subtype classification in the Fudan-HCC cohort.
- Fig. S10. The expression of selected cell marker genes in cell subclusters.
- Fig. S11 The immune microenvironment in EMT-HCC.
- Fig. S12. The interaction loop between tumor cells and fibroblasts.

**Multiplexed immunofluorescence**

The paraffin embedded sections were heated at 60°C for 45 minutes and sequentially immersed in xylene substitute for 2*10 minutes, anhydrous ethanol for 2*3 minutes, 95% ethanol for 2*3 minutes and rinsed under running water for 10 minutes. Sodium citrate antigen repair solution was used to perform antigen repair. 10% BSA was used to block the tissue for 1 hour at room temperature. The tissue was incubated with the appropriate primary antibody for 1 hour at room temperature and washed with TBST buffer for 3*3 minutes. The tissue was incubated with secondary antibody for 10 minutes at room temperature and washed with TBST buffer for 3*3 minutes. The tissue was incubated with fluorescent stain amplified signal solution at room temperature with for 10 minutes and washed with TBST buffer for 3*3 minutes. Sodium Citrate Antigenic Repair solution was used to repair the antigen again. To this point, the first round of staining was completed, and the above process was repeated for a new round of staining. After all the target proteins were stained, the tissue was incubated with DAPI dye for 5 minutes at room temperature and then washed with TBST buffer for 3*3 minutes. Anti-fluorescence quenching blocking agent was used to block the slide. Staining results were observed and analysed under a fluorescence microscope. All antibodies used in this experiment (anti-S100A6 antibody, ab181975; anti-ARG1 antibody, ab133543; anti-Topoisomerase II alpha antibody, ab52934; anti-TGF-β antibody, ab170874; anti-TGF-βR antibody, ab51871; anti-CTGF antibody, ab5097; anti-FAP antibody, ab314456) were purchased from Abcam company

**Flow cytometry**

HCC cell lines (PLC, Hep3B, 97-H, LM3) were digested with trypsin-EDTA (25200072, Gibco) and resuspended in PBS (10010023, Gibco). Next, we using True-Nuclear™ Transcription Factor Buffer Set (424401, BioLegend) to penetrate the nuclear membrane. Then, cells were labelled with 488-conjugated S100A6 antibody (CL488-10245, Proteintech), Brilliant Violet 421 anti-human Ki-67 (350505, BioLegend), and PE anti-human Arginase I (369703, BioLegend) for 30 min at 4°C. After washing with PBS, the samples were filtered using a 40 mm strainer to obtain single cell suspension. The samples were analysed using FlowJo (v10.8, TreeStar) after detection on Sony SH800S instrument.

**Histopathological analysis and immunohistochemistry Staining**

10% formalin was used to fix human and mouse tissue used in this research. The fixed tissue was embedded in paraffin and cut into 4-μM-thick sections for subsequent testing. Hematoxylin and eosin (HE) reagent was used to stain. Histopathological changes were completed by experienced pathologists. Immunohistochemistry analysis was performed as following. In short, the tissue sections were incubated with appropriate primary antibodies overnight before incubation with the corresponding secondary antibodies. DAB Horseradish Peroxidase Color Development Kit (Beyotime, Shanghai, China) was used at room temperature to visualize the stained sections. The images were observed under an inverted microscope. ImageJ software (Media Cybernetics, Silver Springs, MD, USA) was used to evaluate the quantitative results. All antibodies used in this experiment (anti-CD8 alpha antibody, ab237709; anti-CD56 antibody, ab313779; anti-CD4 antibody, ab133616; anti-FOXP3 antibody, ab20034; anti-FAP antibody, ab314456) were purchased from Abcam company.

**siRNA transfection**

Cells were seeded in 24-well plates at a ratio of 0.5-2.0*10^5^/well. Cells were transfected when grown to 70-90% confluence. Opti-MEM™ medium **was used to dilute** Lipofectamine™ 3000 Reagent and siRNA. The solution of Lipofectamine™ 3000 and siRNA was mixed at a ratio of 1:1. This mixed solution was incubated at room temperature for 10-15 min and add to cell culture. Cells were incubated at 37°C for 2 days and then used to future experiment. The siRNA sequences involved in this research are as follows:

| Name | strand | Sequence(5’-3’) |
| --- | --- | --- |
| SPP1-human-1 | sense sequence | GTTTCACAGCCACAAGGAC |
|  | antisense sequence | GTCCTTGTGGCTGTGAAAC |
| SPP1-human-2 | sense sequence | GCGAGGAGTTGAATGGTGCATACAA |
|  | antisense sequence | TTGTATGCACCATTCAACTCCTCGC |
| SPP1-human-3 | sense sequence | CGACTCTGATGATGTAGATGACACT |
|  | antisense sequence | AGTGTCATCTACATCATCAGAGTCG |
| CD44-human-1 | sense sequence | CCAUCUGUGCAGCAAACAATT |
|  | antisense sequence | UUGUUUGCUGCACAGAUGGTT |
| CD44-human-2 | sense sequence | CAGAAAGGAGAAUACAGAATT |
|  | antisense sequence | UUCUGUAUUCUCCUUUCUGTT |
| CD44-human-3 | sense sequence | GCAGGUAUGGGUUCAUAGATT |
|  | antisense sequence | UCUAUGAACCCAUACCUGCTT |
| control | sense sequence | UUCUCCGAACGUGUCACGUTT |
|  | antisense sequence | ACGUGACACGUUCGGAGAATT |

**Cell co-culture**

Fibroblast cell line (LX2) and HCC cell lines (PLC/PRF/5, Hep3B, MHCC97-H, MHCC-LM3) used in this research were provided by Department of Liver Surgery & Transplantation, Liver Cancer Institute, Zhongshan Hospital, Fudan University). All the cell lines were identified by short tandem repeat typing and mycoplasma detection kit (Shanghai GeneChem Co) was used to exclude mycoplasma contamination. These cells were cultured with RPMI 1640 medium containing 10% FBS in a sterile cell culture incubator at 37°C with 5% CO_2_. When the cells grew to 90% confluence, fresh RPMI 1640 medium containing 10% FBS was replaced, and the supernatant of the cells (conditioned medium, which was rich in various cytokines secreted by the cells) was extracted every 2 days and frozen in -80°C for future use. LX2 cells were stimulated with conditioned medium of HCC cell lines (PLC-CM, HepG2-CM, 97H-CM, LM3-CM) to observe the pathophysiological effects on LX2 cell. To verify whether SPP1 could induce LX2 cells to differentiate into tumor-associated fibroblasts (CAFs), we stimulated LX2 cells with SPP1 human recombinant protein (rhSPP1, R&D Systems, No.1433-OP), while adding SiRNA to inhibit CD44 expression to block SPP1 from binding of target. During cell culture, we also collected the conditioned medium secreted by LX2 after stimulation with rhSPP1(activated LX2-secreted conditioned medium, ^ACT^LX2-CM) and used it to culture PLC cells to verify the regulatory effect of CAFs on tumor cells. Recombinant human TGF-β (rhTGF-β, R&D Systems, No.246-LP) and CCN2 protein (rhCCN2, R&D Systems, No.9190-CC) and CCN2 antagonist (FG-3019, MedChemExpress, HY-P99288) were also used to stimulate PLC cells to observe the changes of tumor cells.

**Transwell assay**

A 24-well transwell chamber (8.0 µm pore size; Corning) was used to determine the migratory capacity of LX2 cells and the migratory and invasive capacity of PLC cells. Briefly, LX2 or PLC cells were inoculated in the upper compartment of the transwell chamber and added to RPMI 1640 medium without FBS. RPMI 1640 medium containing 10% FBS was used as a chemoattractant in the lower compartment. PLC-CM, HepaG2-CM, MHCC97-H-CM, HCCLM3-CM, siSPP1, and Bivatuzumab were added to the upper compartment to stimulate the LX2 cells to observe the changes in their migratory ability, respectively. LX2-CM, ^ACT^LX2-CM, rhTGF-β, rhCCN2, and FG-3019 were added to the upper compartment to stimulate PLC cells to observe changes in their migration and invasion abilities. After 24 hours of stimulation, cells that crossed the membrane pores were fixed with 4% paraformaldehyde and stained with 0.5% crystal violet reagent. After washing with 1x phosphate-buffered saline(PBS, 10010023, Gibco), images were taken using a light microscope (100×magnification; Olympus Corporation, Tokyo, Japan). Five fields of view were randomly selected, and the average cell number was determined.

**Cell proliferation inhibition assay**

We used two methods to inhibit the proliferation of PLC cells. In the first type, we added 1ug/ml of mitomycin C(M476633, aladdin) to the culture medium of PLC cells. After stimulation for one hour, cells were washed with PBS and replaced with complete medium. In the second, after washing the PLC cells with PBS, the culture conditions were changed to medium without fetal bovine serum(FBS) to inhibit cell proliferation.CCK8(C0037, Beyotime Biotechnology) assay was used to detect the cell growth rate.

**Western blotting**

The total protein of cell lines used in this research (PLC, Hep3B, 97H, LM3, LX2 cell) was extracted using RIPA Lysis Buffer (Beyotime, Shanghai, China). BCA Protein Assay Kit (Beyotime, Shanghai, China) was used to detect protein concentration. 20μg protein samples was loaded onto 10% or 12.5% SDS-PAGE and transferred to the nitrocellulose membrane (Beyotime, Shanghai, China). Skimmed milk was used to block the membrane. Primary and secondary antibodies were used to incubate the membrane. Omni-ECL™ Femto Light Chemiluminescence Kit (Epizyme Biotech, Shanghai, China) was used to detected expression of protein. Anti-FAP (66562s, CST), anti-SPP1 (ab219056, Abcam), anti-S100A6 (ab181975, Abcam), anti-N-cadherin (13116, CST), anti-E-cadherin (ab314063, Abcam), anti-vimentin(A19607, Abclonal), anti-Smad3 (9523, CST), anti-Phospho-Smad3 (9520, CST), anti-CD44 (A12410, Abclonal), anti-β-Actin (4967, CST) and HRP-labeled Goat anti-Rabbit IgG (ab6721, Abcam) were purchased from Cell Signaling Technology (USA), Abcam, or Abclonal company.

**ELISA**

Firstly, adding 50μl sample solution to the test wells, 500ul working solution was added and the test wells were incubated at 37°C for 1.5 hours. After washing the test wells 5 times, 100μl of HRP working solution was added to each well and incubated at 37°C for 1.5 hours. Then, washing the test wells 3 times, and 90μl of substrate solution was added and incubated for 15 minutes at 37°C without light, followed by the addition of 50μl of termination solution. The OD value of the test wells at 450nm was measured within 5 minutes. The concentration of the sample to be measured was calculated under the standard curve.

**Metastatic model based on tail vein injection**

Six-week-old male BALB/c mice were ordered from Shanghai Model Organisms Center maintained under specific pathogen-free housing with a maximum of five mice per cage. The experiments were performed following the institutional guidelines strictly and were approved by the Institutional Animal Care and Use Committee of the Shanghai Model Organisms Center (2019-0011).

1×10^6^ PLC cells resuspended in 200μL PBS (10010023, Gibco) were injected into 6-week-old nude mice by tail vein injection to observe the formation of distant metastasis induced by HCC cells. To investigate the effect of the CCN2/TGF-β-Smad3 signaling pathway on metastasis formation, we injected ^ACT^LX2-CM and FG-3019(HY-P99288, MCE) intraperitoneally singly or in combination twice a week. 12 weeks after cells injection, mice were sacrificed and fresh lung tissues were taken for histopathological examination.

**Metastatic model based on tumor transplantation**

200μL of PBS solution was used to resuspend 1*10^6^ PLC cells (successfully transfected with Luciferase gene in vitro) single or mixed with 5*10^4^ ^ACT^LX2 cells. Cell solution was then injected into the subcutaneous tissue of 6-week-old nude mice. After tumor formation, the tumor tissue was removed and rinsed twice with saline. The viable tumor tissue was selected and cut into small pieces with a diameter of about 1.5 mm. Nude mice were routinely anaesthetised intraperitoneally, fixed in supine position and the skin was disinfected. The skin was cut along the midline of the abdomen to expose the liver lobe. A tunnel of about 3 mm depth was gently poked in the middle part of the left lobe of the liver, and a cotton swab was applied to stop bleeding. The prepared tissue was carefully delivered into the hepatic peritoneum. The wound will be sutured after carefully checking for no active bleeding. Follow the steps above, we transplanted PLC tumors and PLC/^ACT^LX2 mixed tumors into livers of nude mice, respectively. We injected FG-3019 intraperitoneally 2 times per week for 12 weeks into nude mice with mixed PLC/^ACT^LX2 tumors to observed its effect on tumor growth and lung metastasis. During the injection period, bioluminescence images was used to observe tumor growth. 12 weeks after injection, mice were sacrificed and fresh lung tissues were taken for observe lung metastasis using bioluminescence images.

**Supplementary Figure legend**

**Fig. S1**

**
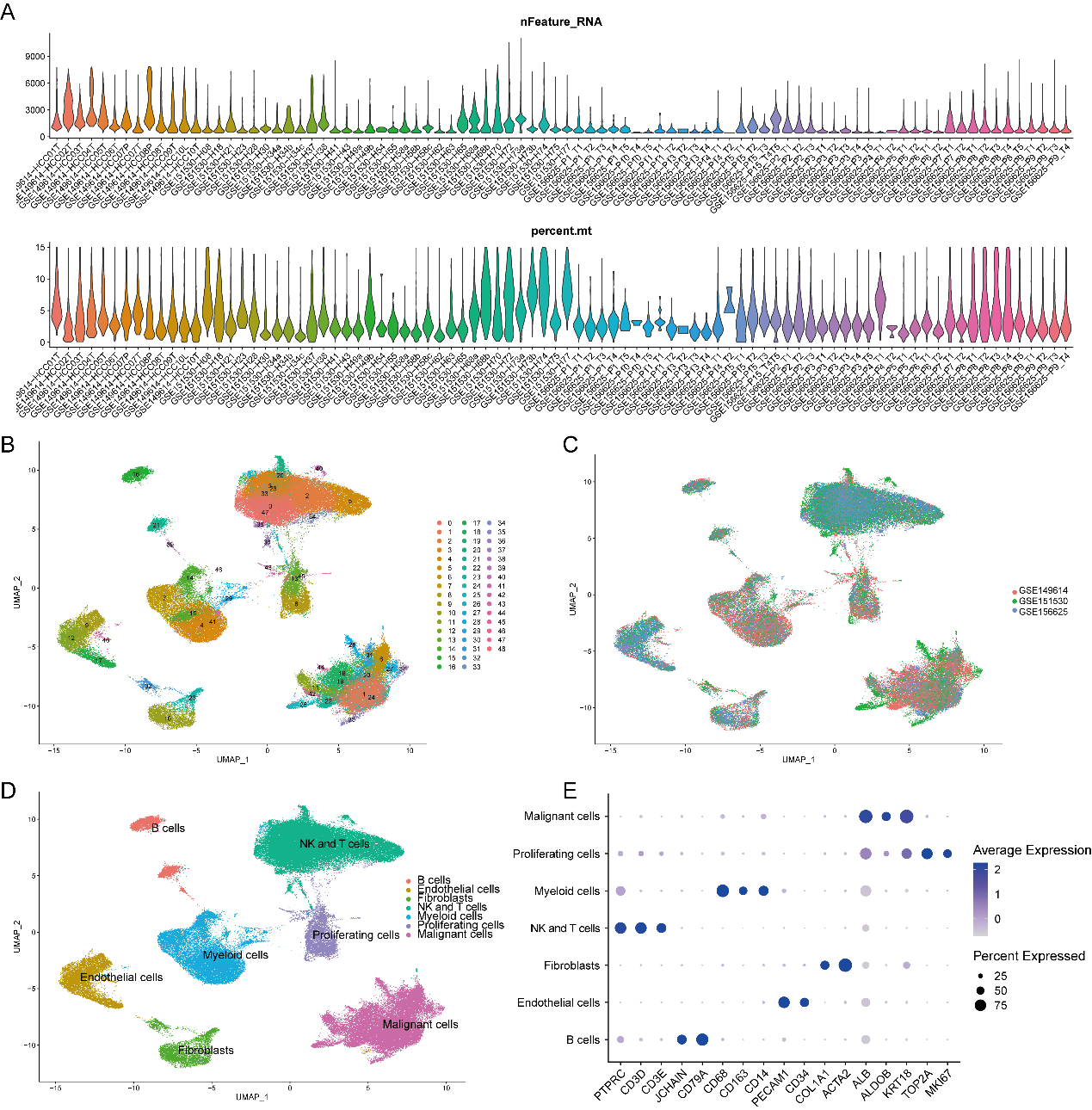
**

**Fig. S1. Identifying malignant cells from integrated single-cell RNA sequencing data.**

(A) The number of features and percentage of mitochondrial gene fraction of included single-cell RNA sequencing data.

(B) The UMAP plot of all cells after quality control.

(C) The UMAP plot showing the distribution of cells from different datasets after mitigating the impact of batch effect.

(D) The UMAP plot showing main cell types.

(E) The dot plot showing the expression of cell marker genes.

**Fig. S2**

**
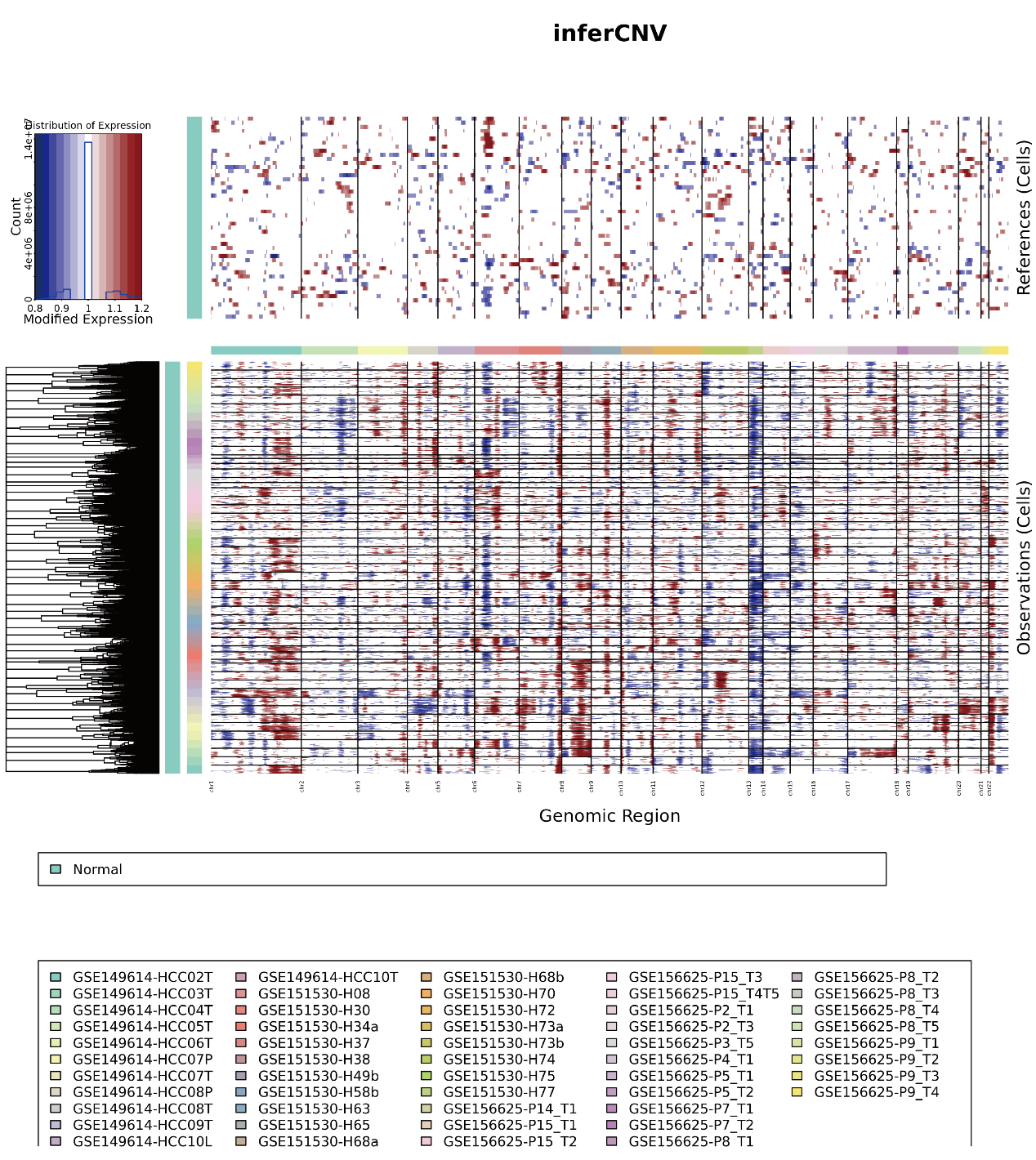
 Fig. S2. The inferred CNV of malignant cells.**

The inferCNV plot showing the inferred CNV score in tumor cells. Stromal cells were used as a normal reference.

**Fig. S3**

**
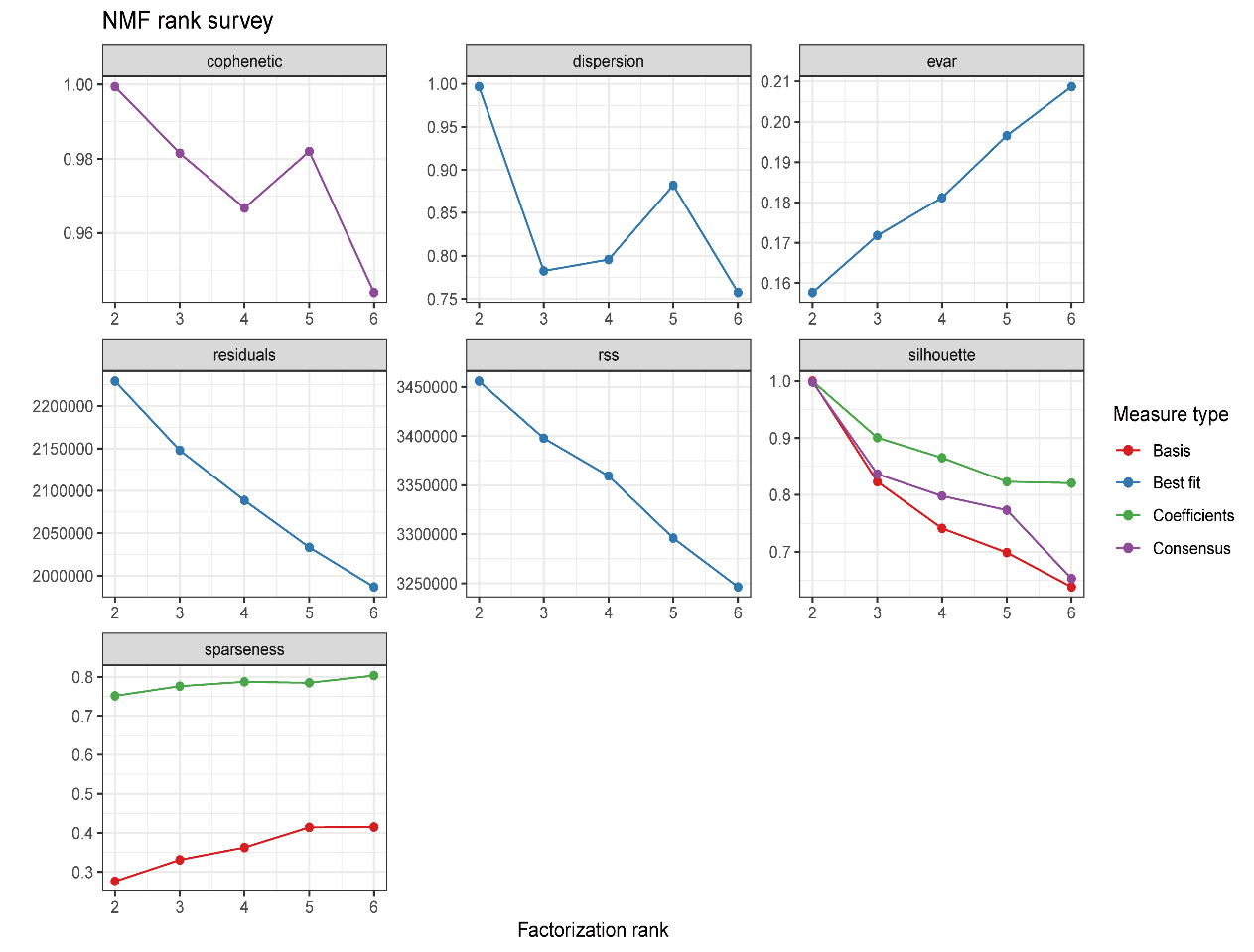
 Fig. S3. The summary of NMF clustering results.**

**Fig. S4**

**
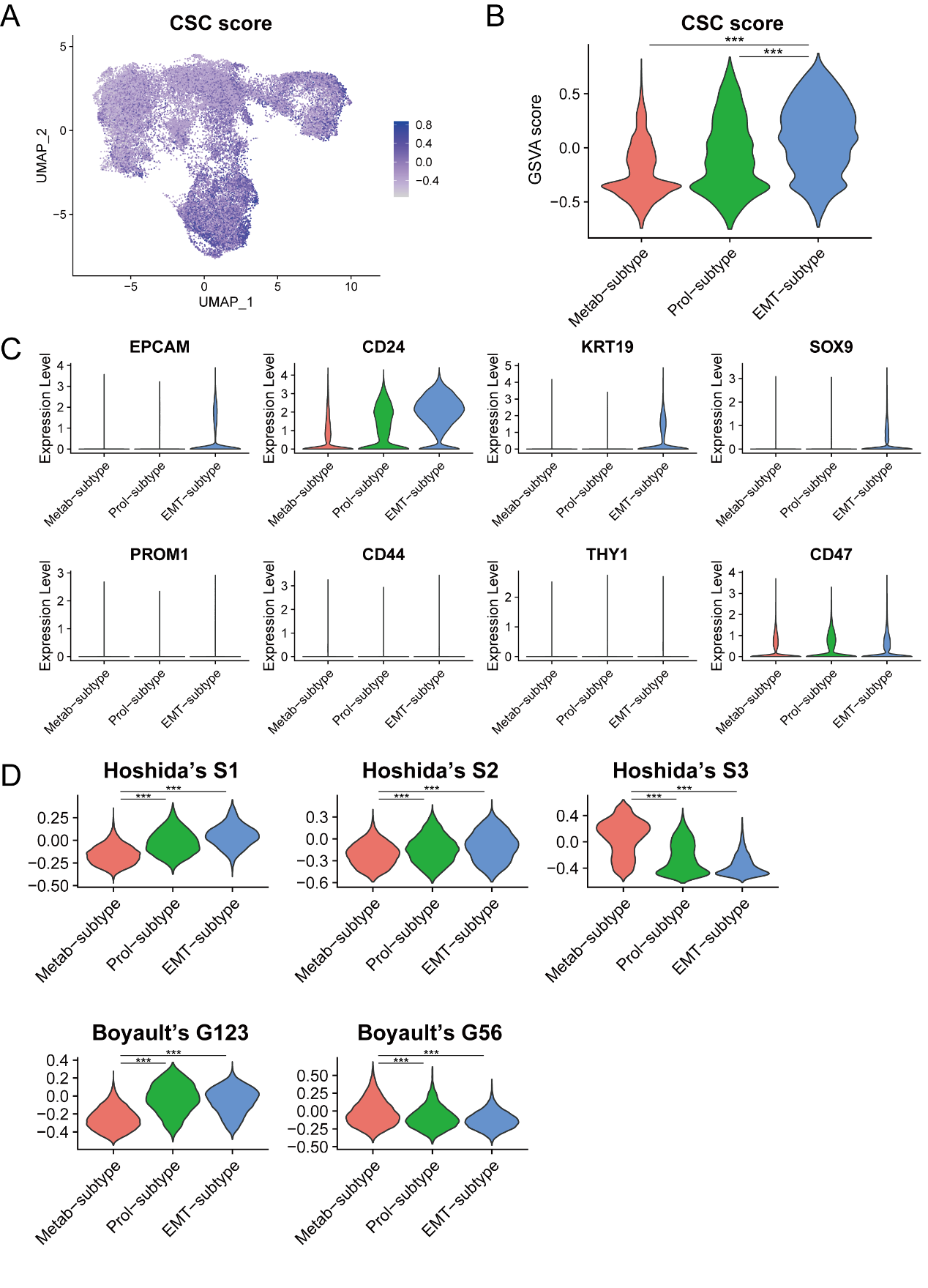
**

**Fig. S4. The distributions of cancer stem cells (CSC) features and bulk-based HCC classifications among the three tumor cell subtypes.**

(A) The UMAP plot showing CSC scores stratified by the three subtypes.

(B) The Violin plot showing CSC scores stratified by the three subtypes.

(C) The Violin plot showing the expression of CSC markers stratified by the three subtypes.

(D) The Violin plot showing the scores of bulk-based HCC classifications stratified by the three subtypes.

**Fig. S5**


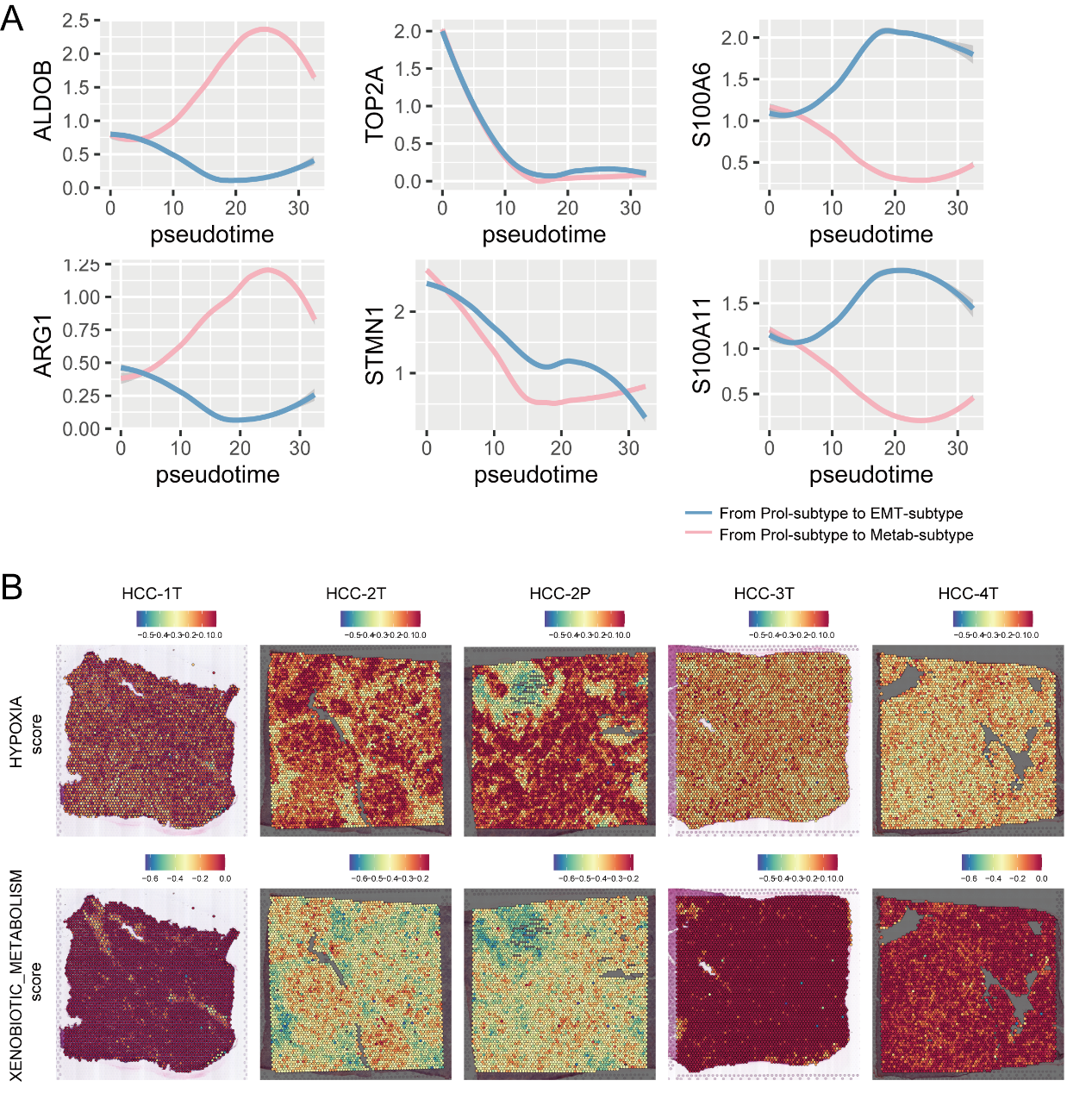


**Fig. S5. The functional features and evolutionary process of the three HCC tumor cell subtypes.**

(A) Expression of selected marker genes along pseudotime from Prol-subtype to Metab-subtype (red line) and from Prol-subtype to EMT-subtype (blue line).

(B) The spatial distribution of selected hallmarks scores in spatial transcriptome data.

**Fig. S6**

**
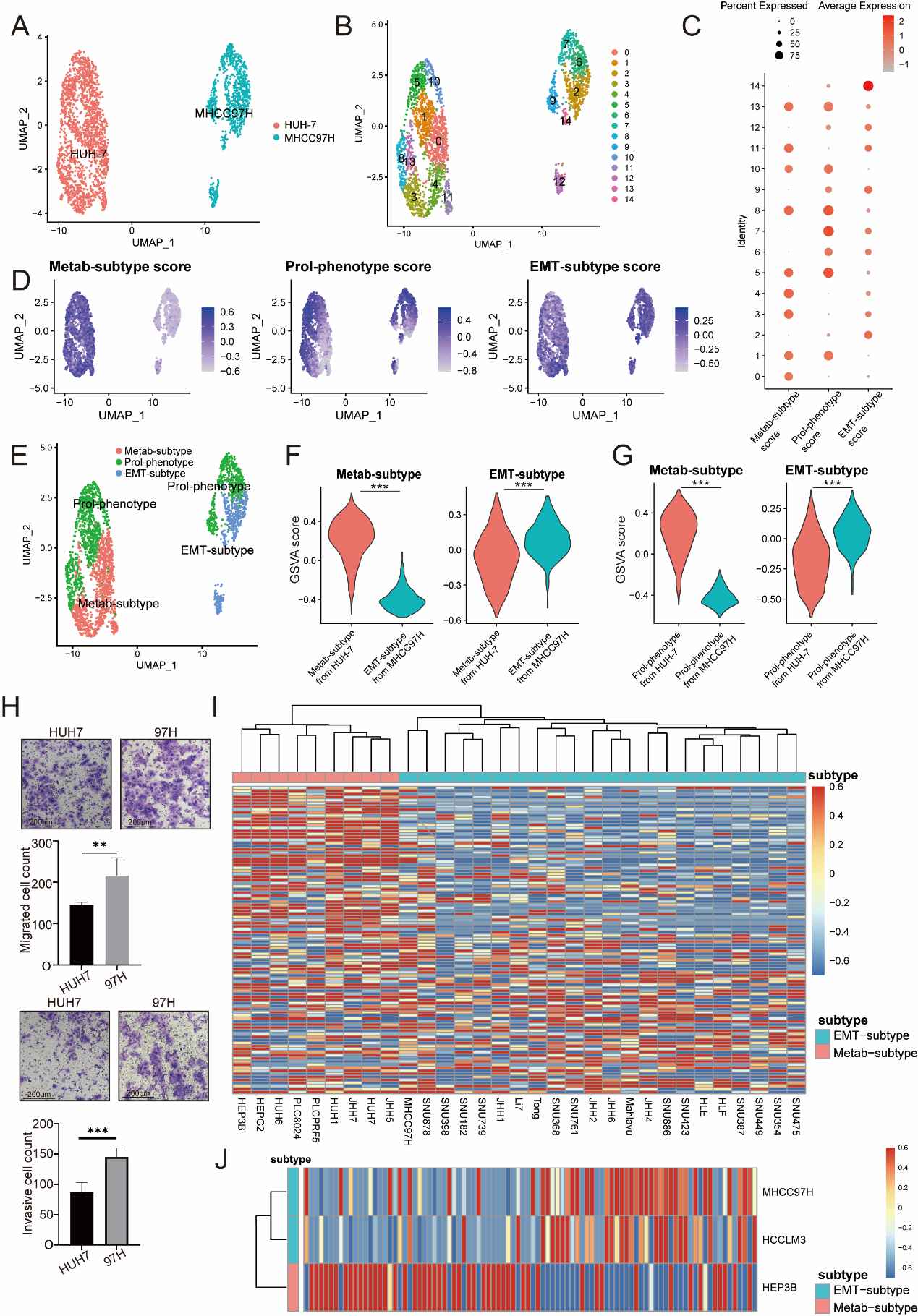
**

**Fig. S6. Validation of the three HCC tumor cell subtypes in cell lines.**

(A). The UMAP plot of HUH7 and 97H cells.

(B). The UMAP plot showing tumor clusters of HUH7 and 97H cells.

(C). The dot plot showing the scores of the three subtypes in HUH7 and 97H cells.

(D). The UMAP plot showing the scores of the three subtypes in HUH7 and 97H cells.

(E). The UMAP plot showing the three subtypes in HUH7 and 97H cells.

(F). The violin plot showing the scores of the Metab-subtype and EMT-subtype in selected HUH7 and 97H cells.

(G). The violin plot showing the scores of the Metab-subtype and EMT-subtype in Prol-phenotype cells of HUH7 and 97H, respectively.

(H). Transwell assay showed invasion and migration capacity of HUH7 and 97H cells.

(I). The heat map showing Metab-subtype and EMT-subtype cell lines in GSE97098.

(J). The heat map showing Metab-subtype and EMT-subtype cell lines in GSE49994.

97H, MHCC97H.

**Fig. S7**


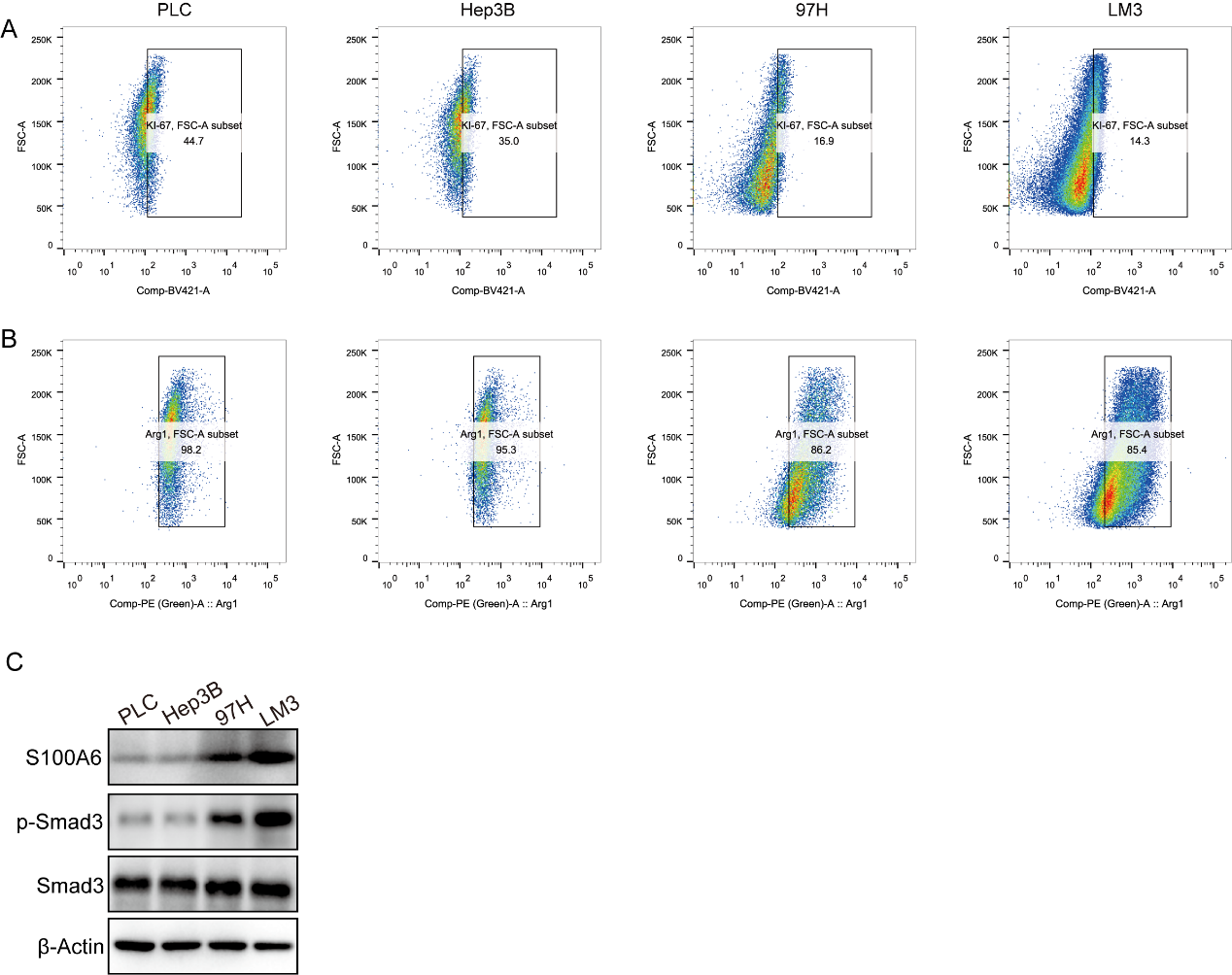


**Fig. S7. The expression of marker genes and translation factors in HCC cell lines.**

(A) Flow cytometry showing KI-67 expression level in four HCC cell lines.

(B) Flow cytometry showing ARG1 expression level in four HCC cell lines.

(C) Western blotting assay showed the expression levels of S100A6 and p-Smad3 in four HCC cell lines.

**Fig. S8**


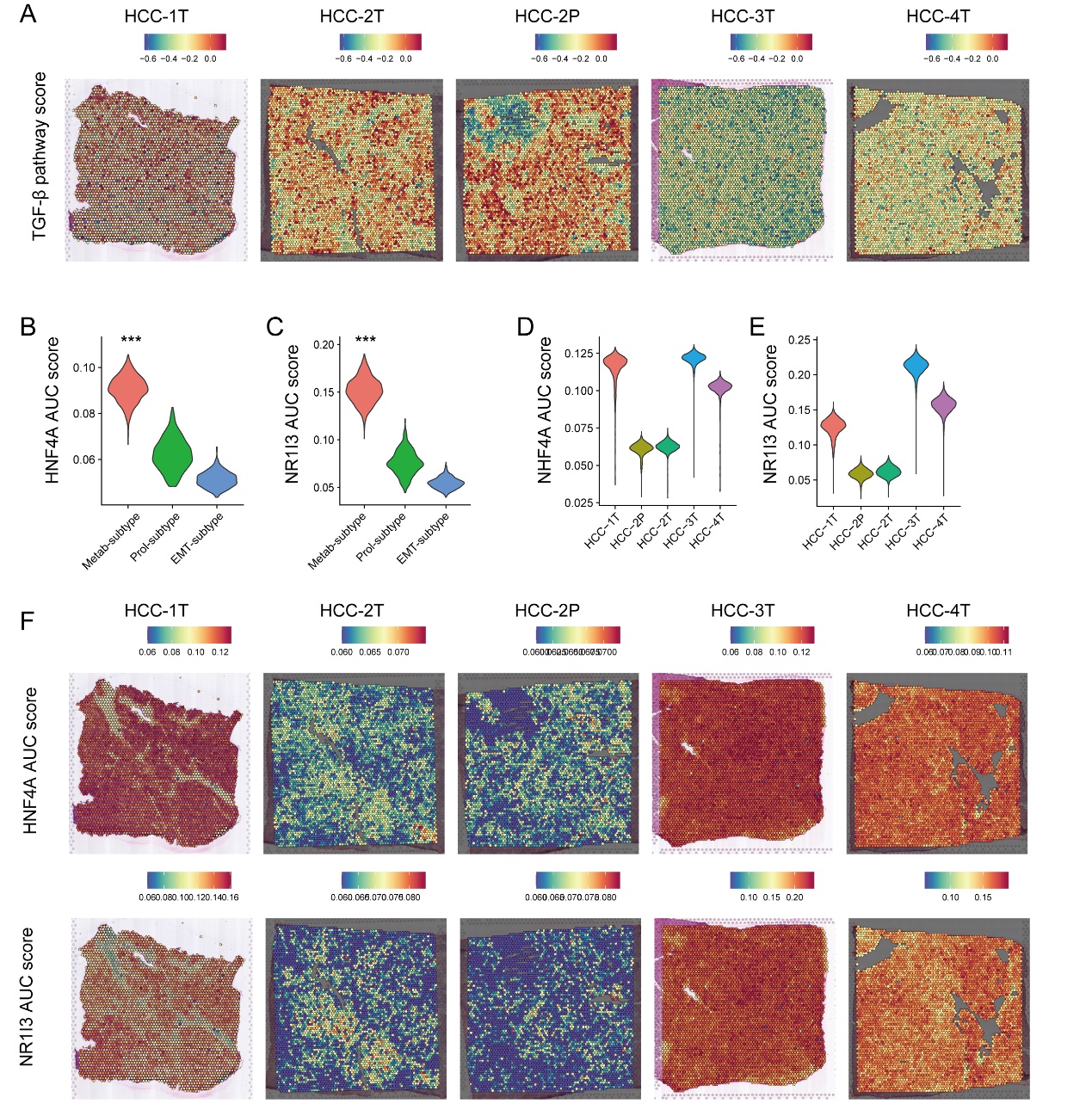


**Fig. S8. The specific activated translation factors and pathways in three subtypes.**

(A) The spatial distribution of TGF-β pathway score in spatial transcriptome data.

(B) HNF4A AUC score stratified by the three subtypes.

(C) NR1I3 AUC score stratified by the three subtypes.

(D) HNF4A AUC score stratified by sample in ST data.

(E) NR1I3 AUC score stratified by sample in ST data.

(F) The spatial distribution of selected translation factors in spatial transcriptome data.

**Fig. S9**


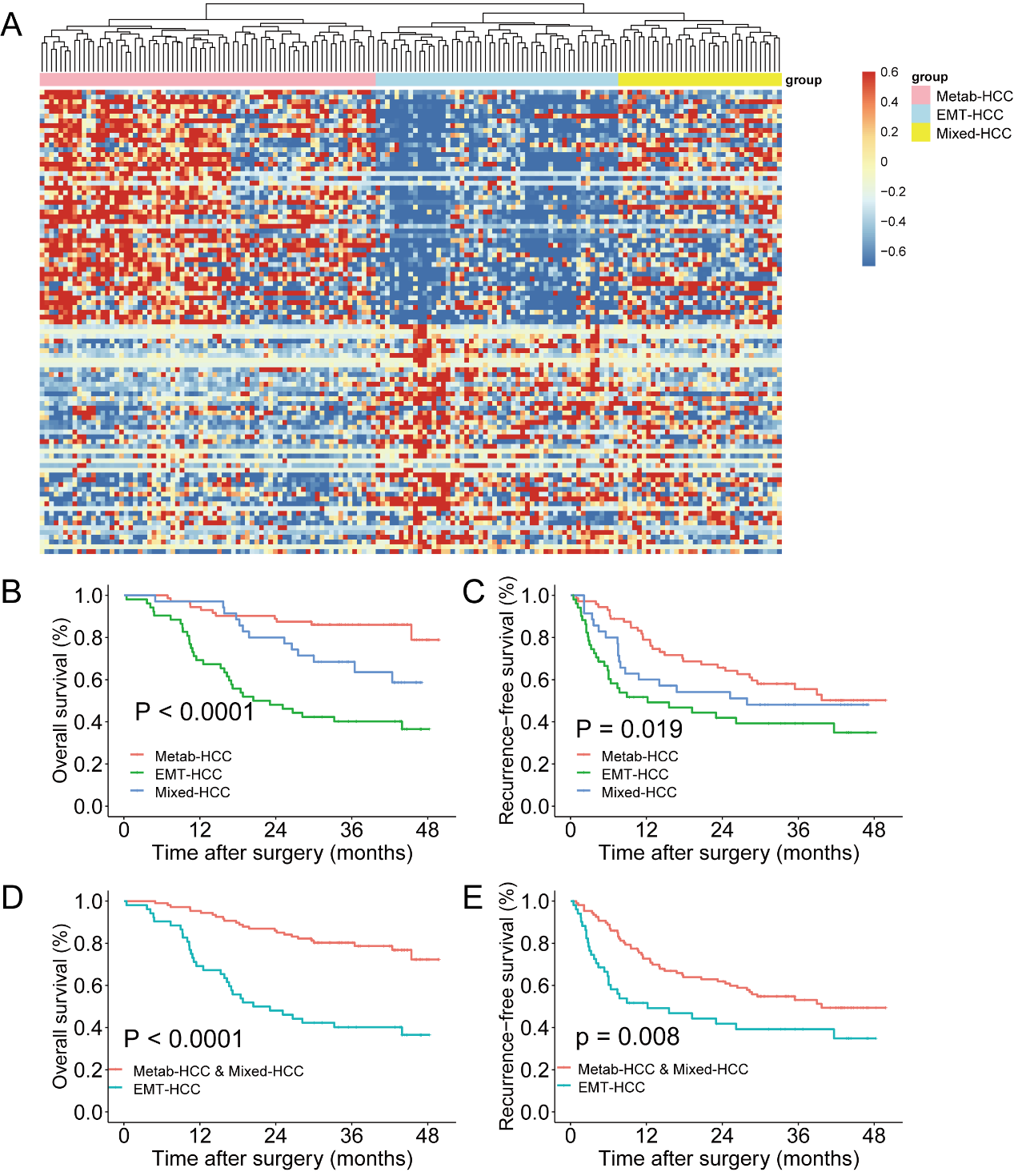


**Fig. S9. Validation of three-subtype classification in the Fudan-HCC cohort.**

(A) The top 50 highly variable genes of Metab-subtype and EMT-subtype divided HCC tumors into three subgroups (Metab-HCC, EMT-HCC, and Mixed-HCC) in the Fudan-HCC cohort.

(B) Kaplan-Meier curves for overall survival (OS) stratified by the three subgroups of HCC in the Fudan-HCC cohort.

(C) Kaplan-Meier curves for recurrence-free survival (RFS) stratified by the three subgroups of HCC in the Fudan-HCC cohort.

(D) Kaplan-Meier curves for OS of EMT-HCC in the Fudan-HCC cohort.

(E) Kaplan-Meier curves for RFS of EMT-HCC in the Fudan-HCC cohort.

**Fig. S10**


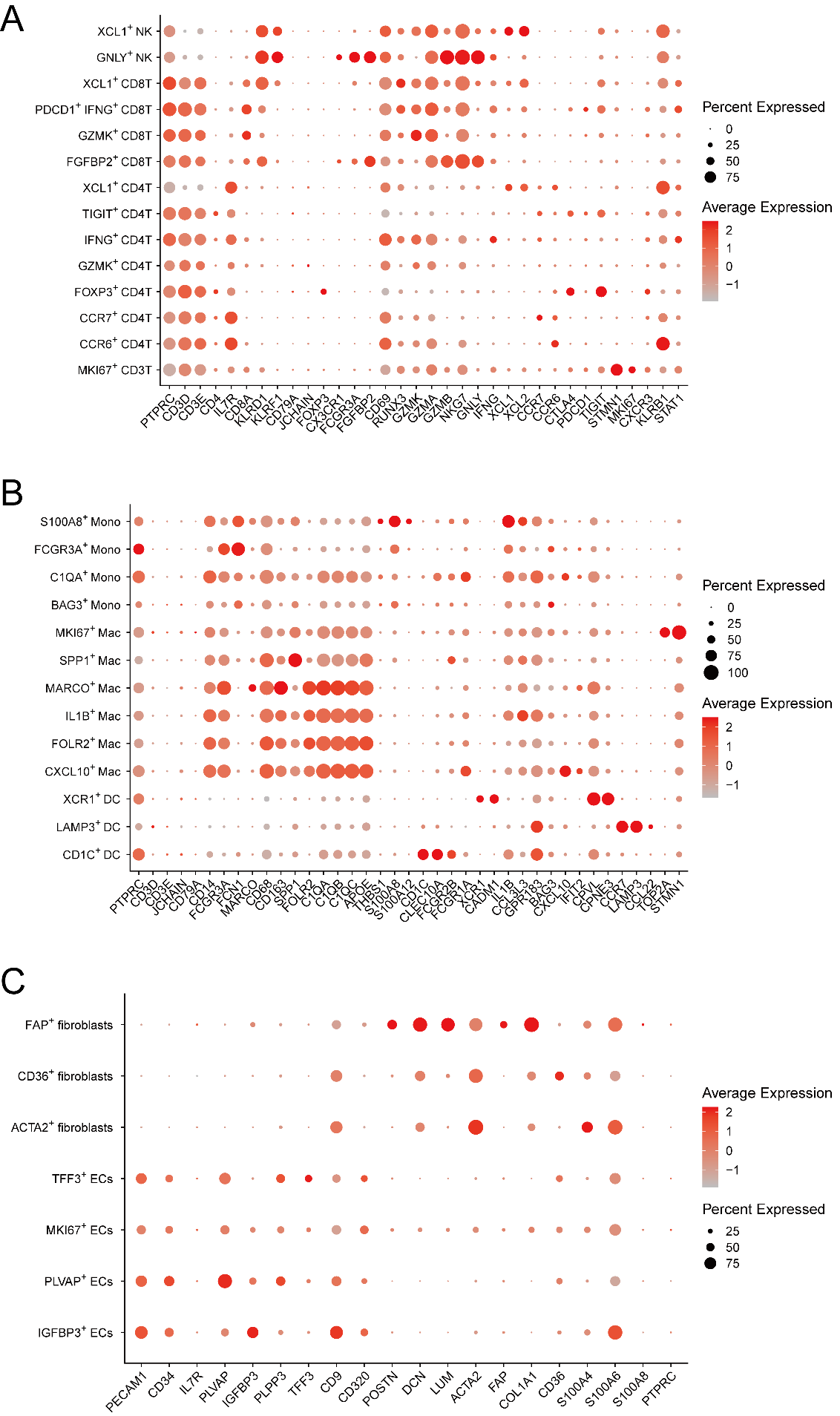


**Fig. S10. The expression of selected cell marker genes in cell subclusters.**

(A) The dot plot showing the expression of selected cell marker genes in NK and T cells.

(B) The dot plot showing the expression of selected cell marker genes in myeloid cells.

(C) The dot plot showing the expression of selected cell marker genes in stromal cells.

**Fig. S11**

**
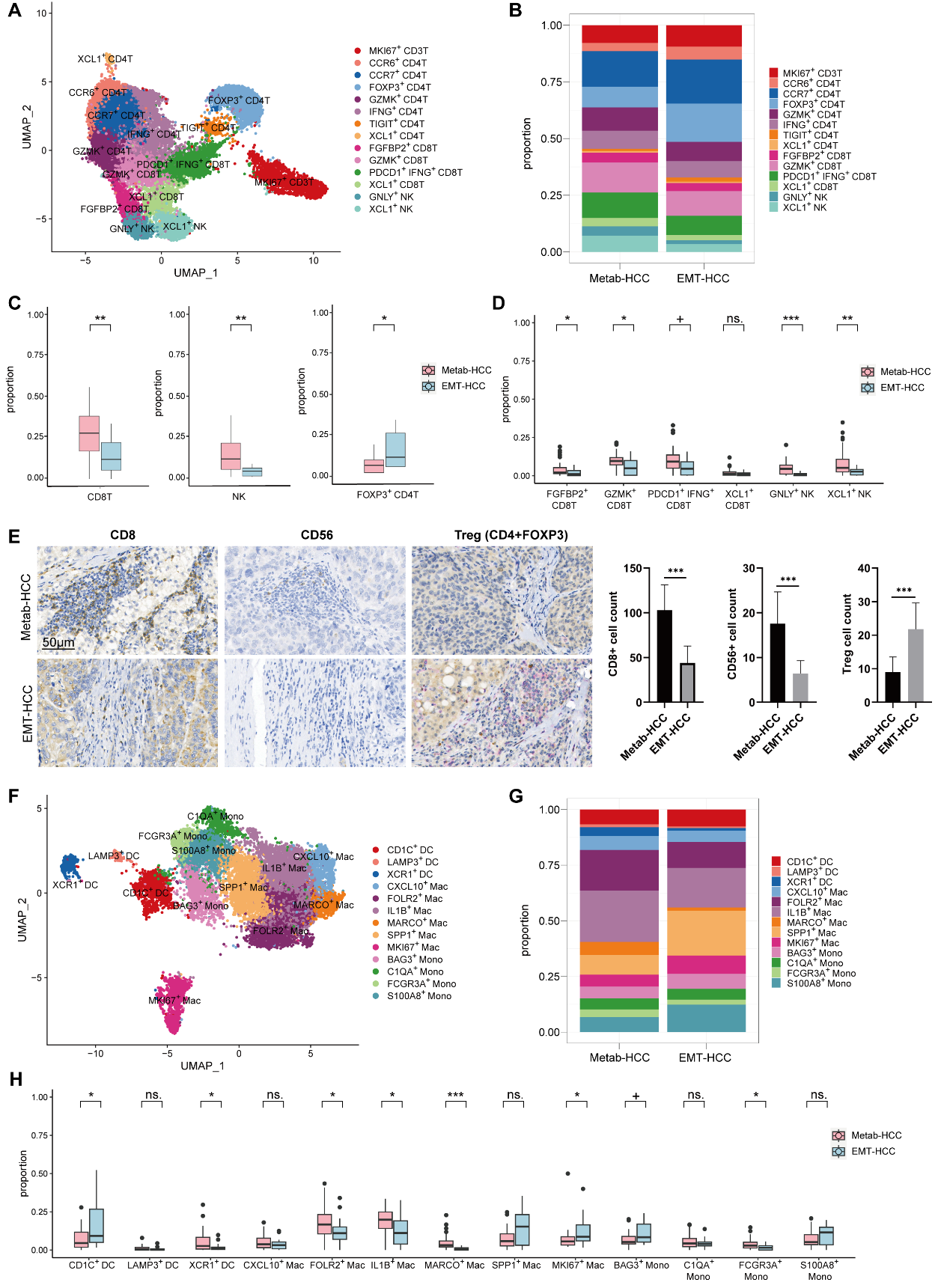
**

**Fig. S11. The immune microenvironment in EMT-HCC.**

(A) The UMAP plot of NK and T cells.

(B) Stacked bar chart showing the compositions of the NK and T subclusters in Metab-HCC and EMT-HCC.

(C) Box plot showing the fraction of CD8^+^ T, NK, and Treg cells in Metab-HCC and EMT-HCC.

(D) Box plot showing the fraction of subclusters of CD8^+^ T and NK cells in Metab-HCC and EMT-HCC.

(E) Immunohistochemistry images showed the number of CD8^+^ T, NK, and Treg cell in Metab-HCC and EMT-HCC.

(F) The UMAP plot of myeloid cells.

(G) Stacked bar chart showing the compositions of the myeloid subclusters in Metab-HCC and EMT-HCC.

(H) Box plot showing the fraction of subclusters of myeloid cells in Metab-HCC and EMT-HCC.

**Fig. S12**

**
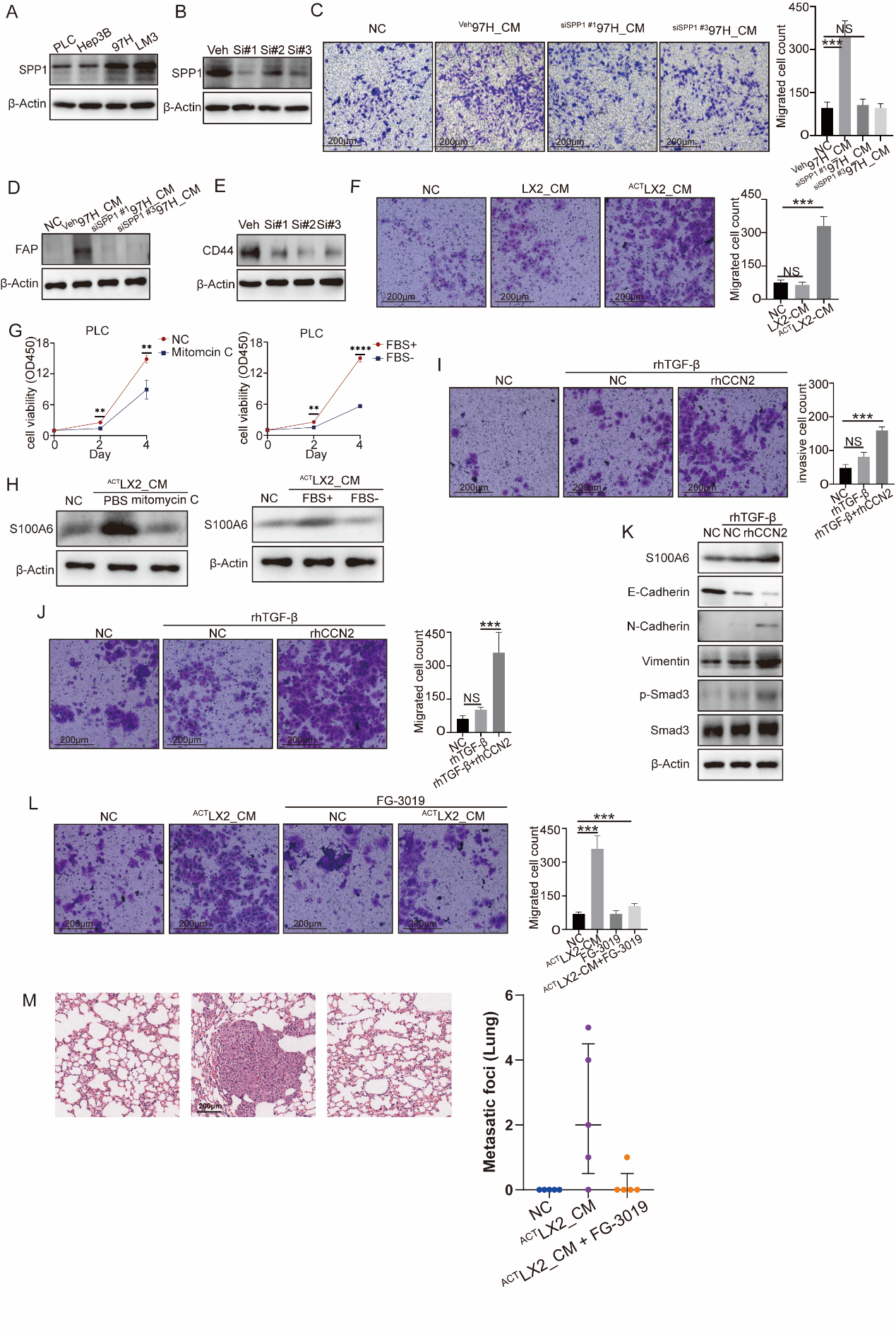
**

**Fig. S12. The interaction loop between tumor cells and fibroblasts.**

1. Western blotting assay showed that SPP1 expression levels were significantly up-regulated in 97H and LM3 cells.
2. Western blotting assay SPP1 expression levels of 97H were decreased while treated with siRNA.
3. Transwell assay showed that knocking down SPP1 in 97H cells (^siSPP1^97H) significantly reversed the enhanced attraction of LX2 cells induced by ^Veh^97H_CM.
4. Western blotting assay showed that knocking down SPP1 in 97H cells (^siSPP1^97H) significantly reversed the enhanced FAP protein level in LX2 cells induced by ^Veh^97H_CM.
5. Western blotting assay CD44 expression levels of LX2 cells were decreased while treated with siRNA.
6. Transwell assay showed that ^ACT^LX2_CM significantly promoted the migration ability of PLC cells.
7. CCK8 assay showed that mitomcin C and deprivation of fetal bovine serum in culture medium significantly inhibited cell proliferation.
8. Western blotting showed that change of S100A6 expression was significantly reversed by treatment with mitomycin C or deprivation of fetal bovine serum in culture medium.
9. Transwell assay showed that rhTGF-β significantly promoted the invasive ability of PLC cells, which was further enhanced by CCN2.
10. Transwell assay showed that rhTGF-β significantly promoted the migration ability of PLC cells, which was further enhanced by CCN2.
11. Western blotting assay showed that rhTGF-β significantly promoted the protein levels of S100A6, N-cadherin, Vimentin and p-Smad3 in PLC cells while decreased expression of E-cadherin, which was further enhanced by rhCCN2.
12. Transwell assay showed that FG-3019 reversed the enhanced migration ability of PLC cells induced by ^ACT^LX2_CM.
13. HE images of lung metastases generated in distant seeding mouse model tail-vein-injected with PLC in the condition of injecting ^ACT^LX2_CM or co-injecting ^ACT^LX2_CM plus FG-3019, n = 5 for each group.

NC, Normal Control; PLC, PLC/PRF/5 cells; 97H, MHCC97H cells; LM3, HCCLM3 cells; CM, conditioned medium; Veh, siRNA control vehicle; siSPP1, SPP1 sensitive siRNA; ^Veh^97H, siRNA control vehicle transfected into 97H cells; ^siSPP1^97H, knocking down SPP1 in 97H cells; ^ACT^LX2_CM, Conditioned medium of SPP1-pretreated LX2 cells; rhSPP1, recombinant human protein SPP1; rhTGF-β, recombinant human protein TGF-β; rhCCN2, recombinant human protein CCN2; FG-3019, Anti-Human CTGF Recombinant Antibody.
