# Supplementary material for: Single-cell tumor heterogeneity landscape of hepatocellular carcinoma: unraveling the pro-metastatic subtype and its interaction loop with fibroblasts
Source: Mol Cancer. 2024 Aug 2;23:157. doi: 10.1186/s12943-024-02062-3 (PMC11295380; doi:10.1186/s12943-024-02062-3)

Figure 6B

FAP

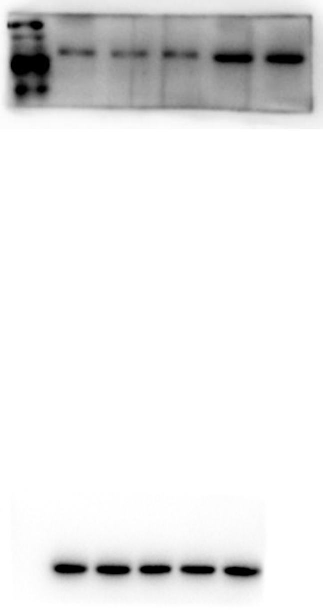

β-Actin

Figure 6D

FAP

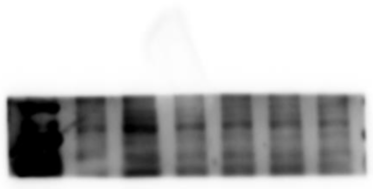

β-Actin

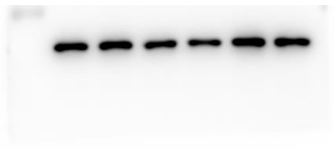

Figure 6F

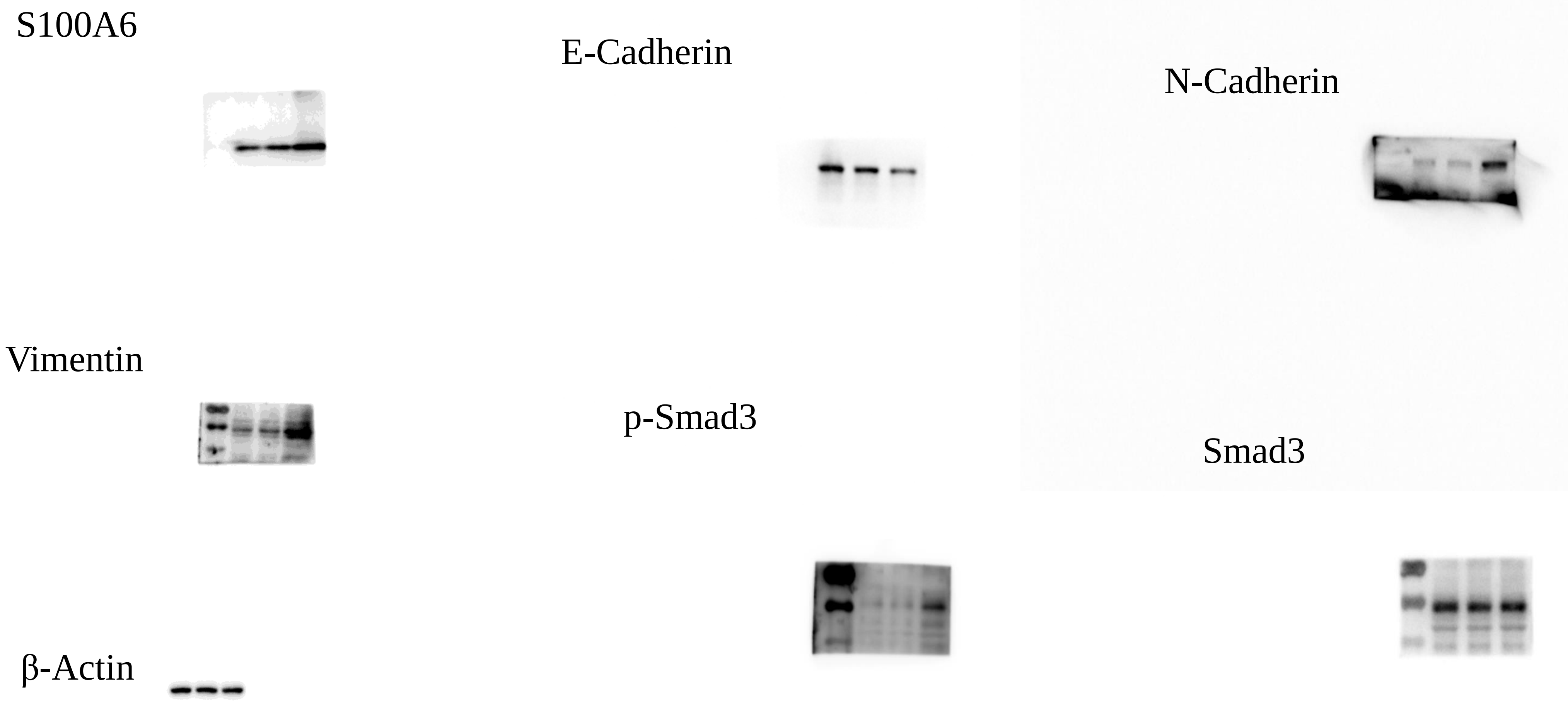

Figure 6K

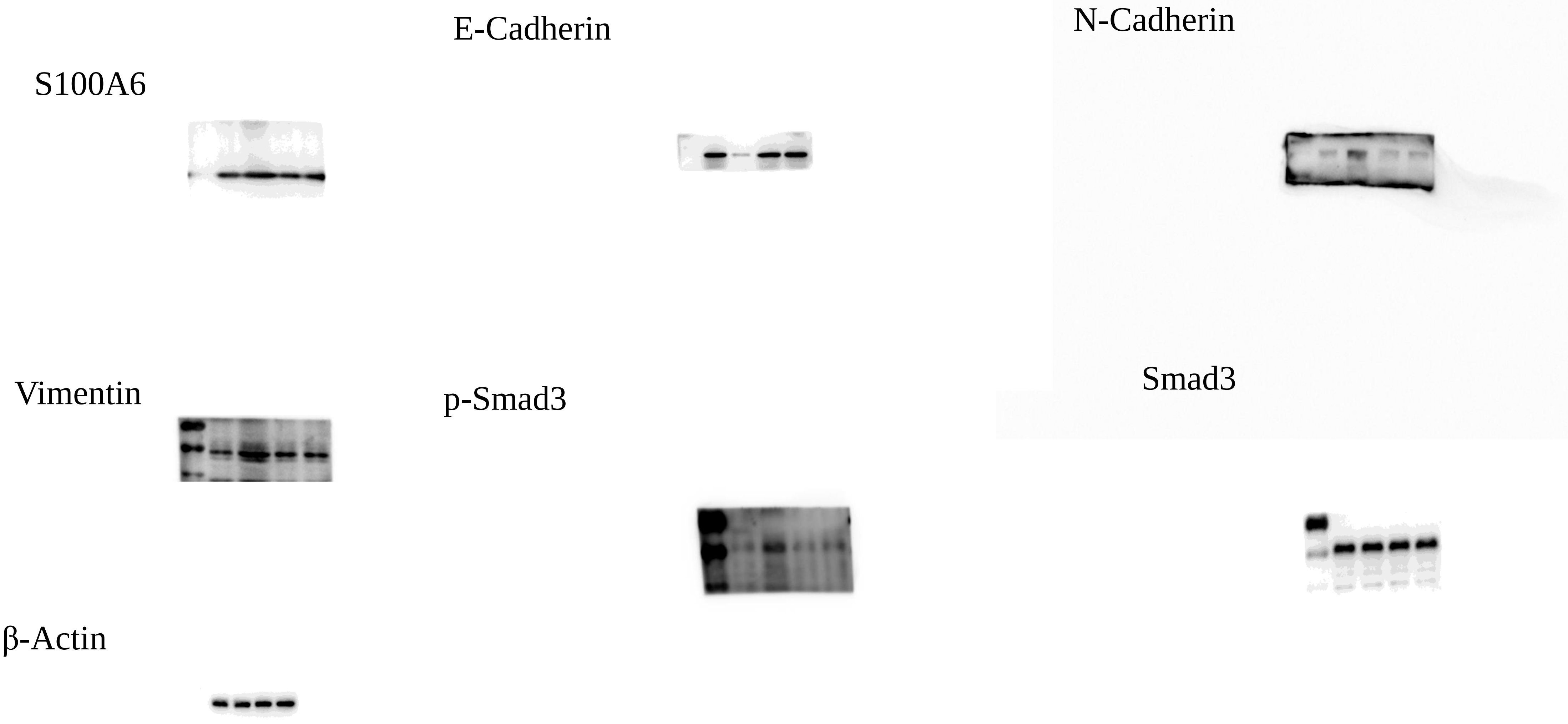

Figure S7C

S100A6

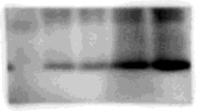

p-Smad3

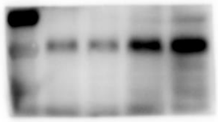

Smad3

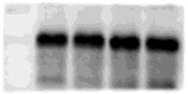

β-Actin

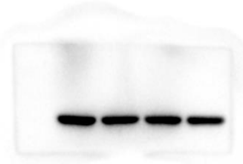

Figure S12A

SPP1

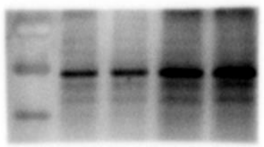

β-Actin

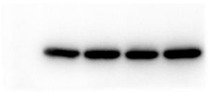

Figure S12B

SPP1

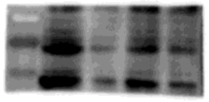

β-Actin

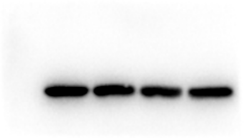

Figure S12D

FAP

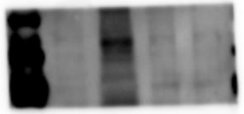

β-Actin

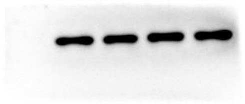

Figure S12E

CD44

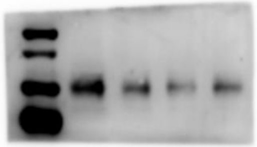

β-Actin

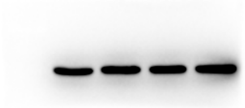

Figure S12H

S100A6

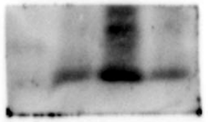

S100A6

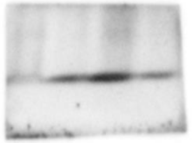

β-Actin

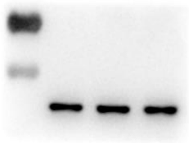

β-Actin

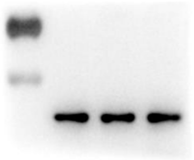

Figure S12K

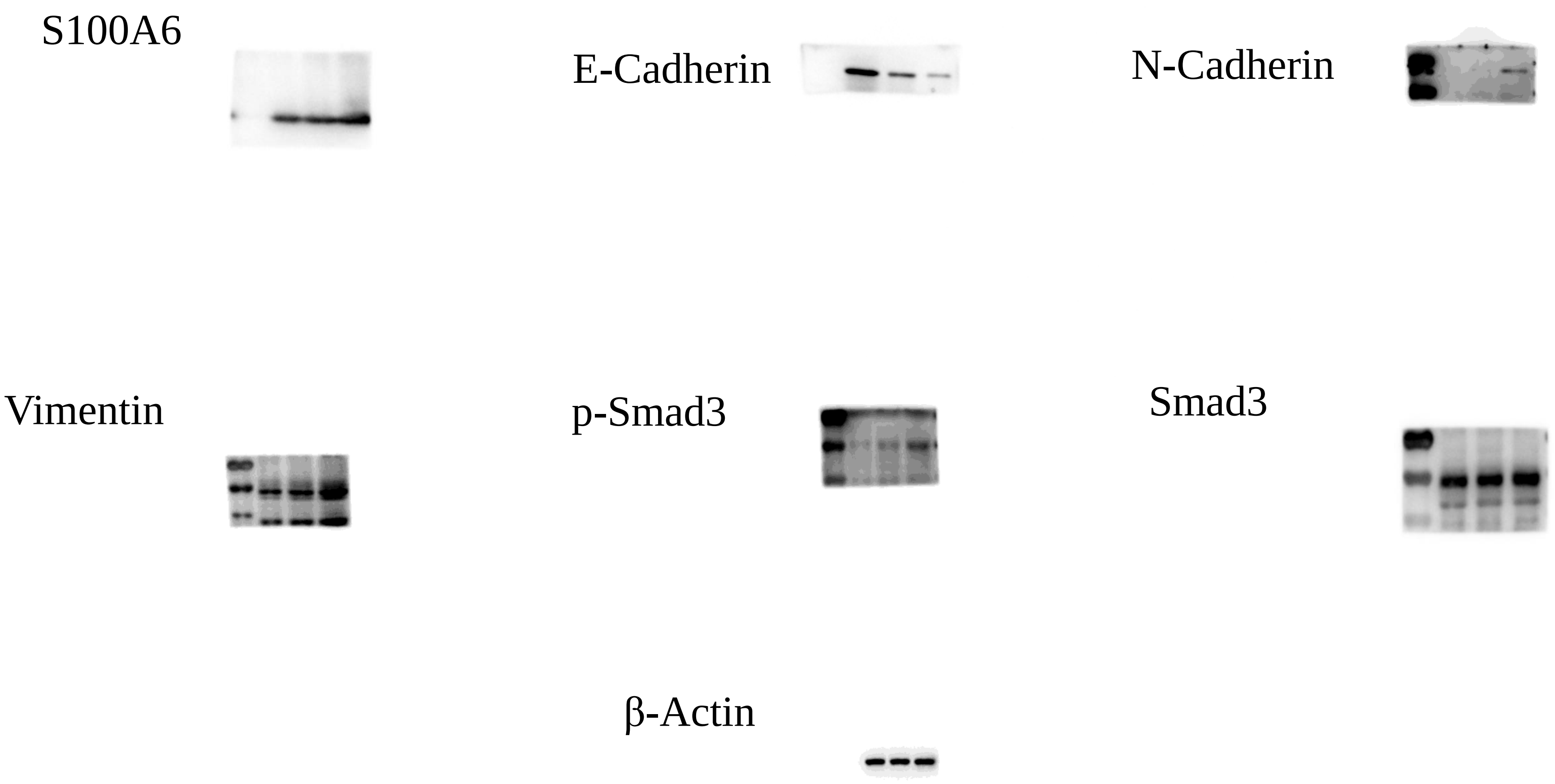

Supplement: Supplementary file 5 — Supplementary Material 5 [file 12943_2024_2062_MOESM5_ESM.pdf]
